# Synthesis and crystal structure of a penta­copper(II) 12-metallacrown-4: *cis*-di­aqua­tetra­kis­(di­methyl­formamide-κ*O*)manganese(II) tetra­kis­(μ_3_-*N*,2-dioxido­benzene-1-carboximidate)penta­copper(II) di­methyl­formamide monosolvate

**DOI:** 10.1107/S2056989020005770

**Published:** 2020-04-30

**Authors:** Gerard P. Van Trieste III, Matthias Zeller, Curtis M. Zaleski

**Affiliations:** a Department of Chemistry and Biochemistry, Shippensburg University, Shippensburg, PA 17257, USA; b Department of Chemistry, Purdue University, West Lafayette, Indiana 479070, USA

**Keywords:** metallacrown, copper complex, salicyl­hydroximate, crystal structure

## Abstract

The title compound, [Mn(C_3_H_7_NO)_4_(H_2_O)_2_][Cu_5_(C_7_H_4_NO_3_)_4_]·C_3_H_7_NO or *cis*-[Mn(H_2_O)_2_(DMF)_4_]{Cu[12-MC_Cu(II)N(shi)_-4]}·DMF, where MC is metallacrown, shi^3−^ is salicyl­hydroximate, and DMF is *N*,*N*-di­methyl­formamide, crystallizes in the monoclinic space group *P*2_1_/*n.* Two crystallographically independent metallacrown anions are present in the structure, and both anions exhibit minor main mol­ecule disorder by an approximate (non-crystallographic) 180° rotation with occupancy ratios of 0.9010 (9) to 0.0990 (9) for one anion and 0.9497 (8) to 0.0503 (8) for the other. Each penta­copper(II) metallacrown contains four Cu^II^ ions in the MC ring and a Cu^II^ ion captured in the central cavity. Each Cu^II^ ion is four-coordinate with a square-planar geometry. The anionic {Cu[12-MC_Cu(II)N(shi)_-4]}^2−^ is charged-balanced by the presence of a *cis*-[Mn(H_2_O)_2_(DMF)_4_]^2+^ cation located in the lattice. In addition, the octa­hedral Mn^II^ counter-cation is hydrogen bonded to both MC anions *via* the coordinated water mol­ecules of the Mn^II^ ion. The water mol­ecules form hydrogen bonds with the phenolate and carbonyl oxygen atoms of the shi^3−^ ligands of the MCs.

## Abstract 2

The title compound [Mn(OH_2_)_2_(C_3_H_7_NO)_4_][Cu_5_(C_7_H_4_NO_3_)_4_]·C_3_H_7_NO or *cis*-[Mn(H_2_O)_2_(DMF)_4_]{Cu[12-MC_Cu(II)N(shi)_-4]}·DMF, where MC is metallacrown, shi^3−^ is salicyl­hydroximate, and DMF is *N*,*N*-di­methyl­formamide, consists of a penta­copper(II) 12-metallacrown-4 anion that is charged-balanced with the *cis*-[Mn(DMF)_4_(OH_2_)_2_]^2+^ cation. In the {Cu[12-MC_Cu(II)N(shi)_-4]}^2−^ anion, all four Cu^II^ ions of the metallacrown ring and the one Cu^II^ ion of the central cavity are four-coordinate with a square-planar geometry. The Mn^II^ counter-cation is six-coordinate with an octa­hedral geometry.

## Chemical context   

Penta­copper(II) 12-metallacrown-4 complexes are ubiquitous in metallacrown (MC) chemistry (Mezei *et al.*, 2007 ▸; Tegoni & Remelli, 2012 ▸; Ostrowska *et al.*, 2016 ▸). A survey of the Cambridge Structural Database (CSD version 5.41, update March 2020; Groom *et al.*, 2016 ▸) reveals that there are 35 different structures; however, even more Cu_5_ 12-MC-4 complexes have been studied in solution to understand the thermodynamic properties of their self-assembly (Mezei *et al.*, 2007 ▸; Tegoni & Remelli, 2012 ▸; Ostrowska *et al.*, 2016 ▸). Initially Cu_5_ 12-MC-4 complexes were only produced with ligands that could form fused five- and six-membered chelate rings such as salicyl­hydroxamic acid or β-amino­hydroxamic acids (Orama *et al.*, 1992 ▸; Gibney *et al.*, 1994 ▸; Halfen *et al.*, 1998 ▸); however, it is now recognized that α- and γ-amino­hydroxamic acids can form Cu_5_ 12-MC-4 complexes that have fused five- and five-membered chelate rings or fused five- and seven-membered chelate rings, respectively (Dallavalle *et al.*, 2001 ▸; Tegoni *et al.*, 2004 ▸, 2007 ▸, 2008 ▸). Penta­copper(II) 12-MC-4 complexes have applications as templates for the assembly of peptide bundles (Cal *et al.*, 2013 ▸), for the sorption of gases and alcohols (Atzeri *et al.*, 2016 ▸; Pavlishchuk *et al.*, 2017 ▸), and as building blocks for one-, two-, and three-dimensional materials (Bodwin & Pecoraro, 2000 ▸; Gumienna-Kontecka *et al.*, 2007 ▸; Lago *et al.*, 2011 ▸; McDonald *et al.*, 2013 ▸; Atzeri *et al.*, 2016 ▸). To date only four other structures have been reported with the metallacrown framework ligand salicyl­hydroxamic acid (H_3_shi): *A*_2_{Cu[12-MC_Cu(II)N(shi)_-4]}, where *A* is either tetra­methyl­ammonium (Gibney *et al.*, 1994 ▸), [Na(15-crown-5)]^+^ (Gibney *et al.*, 1994 ▸), tetra­ethyl­ammonium (Herring *et al.*, 2011 ▸), or tri­ethyl­ammonium (Happ & Rentschler, 2014 ▸). Herein we report the first use of a 3*d* metallic counter-cation to the penta­copper(II) metallacrown: *cis*-[Mn(H_2_O)_2_(DMF)_4_]{Cu[12-MC_Cu(II)N(shi)_-4]}·DMF.
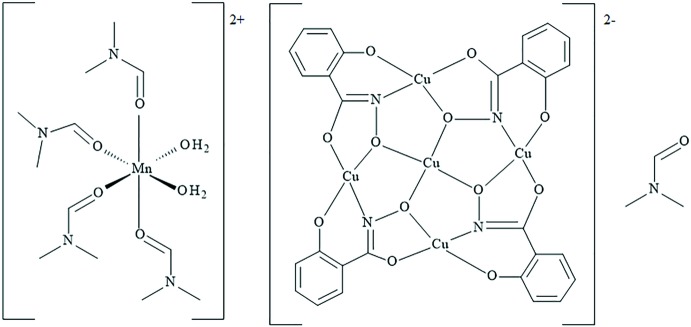


## Structural commentary   

**Figure 1:**
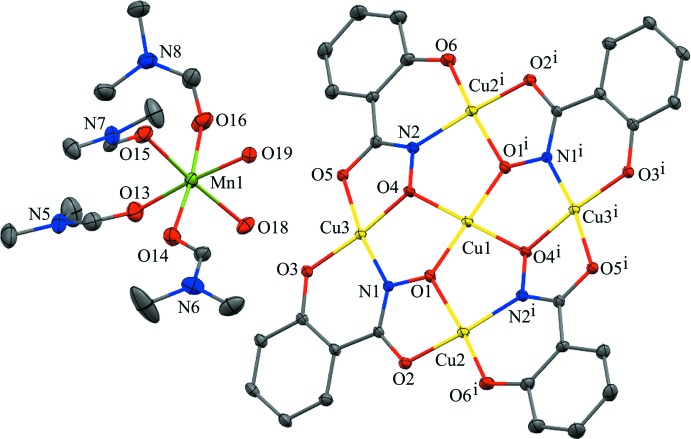
The single-crystal X-ray structure of the ionic pair *cis*-[Mn(H_2_O)_2_(DMF)_4_]{Cu[12-MC_Cu(II)N(shi)_-4]}·DMF associated with Cu1 and with displacement ellipsoids at the 50% probability level [symmetry code: (i) 2 − *x*, −*y*, 1 − *z*]. For clarity, only non-carbon atoms have been labeled, and the MC associated with Cu4, the lattice DMF mol­ecule, H atoms, and disorder have been omitted. Color scheme: yellow – Cu^II^, green – Mn^II^, red – oxygen, blue – nitro­gen, and gray – carbon. All figures were generated with the program *Mercury* (Macrae *et al.*, 2020 ▸).

Two crystallographically independent metallacrown anions are present in the structure, and both are located on crystallographic inversion centers with the central copper ions situated on the inversion center (Figs. 1 ▸ and 2 ▸). Both anions exhibit minor main-mol­ecule disorder by an approximate (non-crystallographic) 180° rotation with an occupancy ratio of 0.9010 (9) to 0.0990 (9) for the anion associated with Cu1 and an occupancy ratio 0.9497 (8) to 0.0503 (8) for the anion associated with Cu4. Thus, only the structures of the main moieties will be discussed. The metallacrowns have an overall square shape as a result of the fused five- and six-membered chelate rings of the salicyl­hydroximate (shi^3−^) ligands, and the MCs are slightly non-planar. In each MC, a copper ion is captured in the central cavity and surrounded by four copper ions of the MC ring. The MC ring has a Cu—N—O pattern that repeats four times to generate the MC central cavity. All five copper ions of each MC are assigned a 2+ oxidation state based on bond-valence-sum (BVS) values (Liu & Thorp, 1993 ▸), average bond length distances, and overall charge-balance considerations (Table 1 ▸). In addition, all five Cu^II^ ions of each MC are four-coordinate, and a SHAPE (*SHAPE 2.1*; Llunell *et al.*, 2013 ▸) analysis of the geometry yields the lowest continuous shape measure (CShM) values for square planar (Table 2 ▸), which is typical for a *d* ^9^ electron configuration (Llunell *et al.*, 2013 ▸; Pinsky & Avnir, 1998 ▸; Casanova *et al.*, 2004 ▸; Cirera *et al.*, 2005 ▸). The coordination environment of the central Cu^II^ ions (Cu1 and Cu4) are composed of four oxime oxygens from four different shi^3−^ ligands. The coordination environments of the ring Cu^II^ ions (Cu2, Cu3, Cu5, and Cu6) consist of *trans* five- and six-membered chelate rings: each five-membered chelate ring is formed by the carbonyl oxygen atom and the oxime oxygen atom of a shi^3−^ ligand, and each six-membered chelate ring is formed by the phenolate oxygen atom and oxime nitro­gen atom of a different shi^3−^ ligand.

The use of the four trianionic shi^3−^ ligands and five divalent Cu^II^ ions yields an MC with overall charge of 2−, {Cu[12-MC_Cu(II)N(shi)_-4]}^2−^. This charge is balanced by the presence of a manganese cation in the lattice: *cis*-[Mn(H_2_O)_2_(DMF)_4_]^2+^. The manganese ion is assigned an oxidation state of 2+ based on the average bond length of 2.171 Å, a BVS value of 2.01 valence units (v.u.), and overall charge-balance considerations (Table 1 ▸). A SHAPE analysis confirms the octa­hedral geometry of the cation (Table 3 ▸). The coordination environment of the Mn^II^ ion consists of four DMF mol­ecules and two water mol­ecules in a *cis* configuration. Lastly, a DMF mol­ecule is located in the lattice.

## Supra­molecular features   

**Figure 3:**
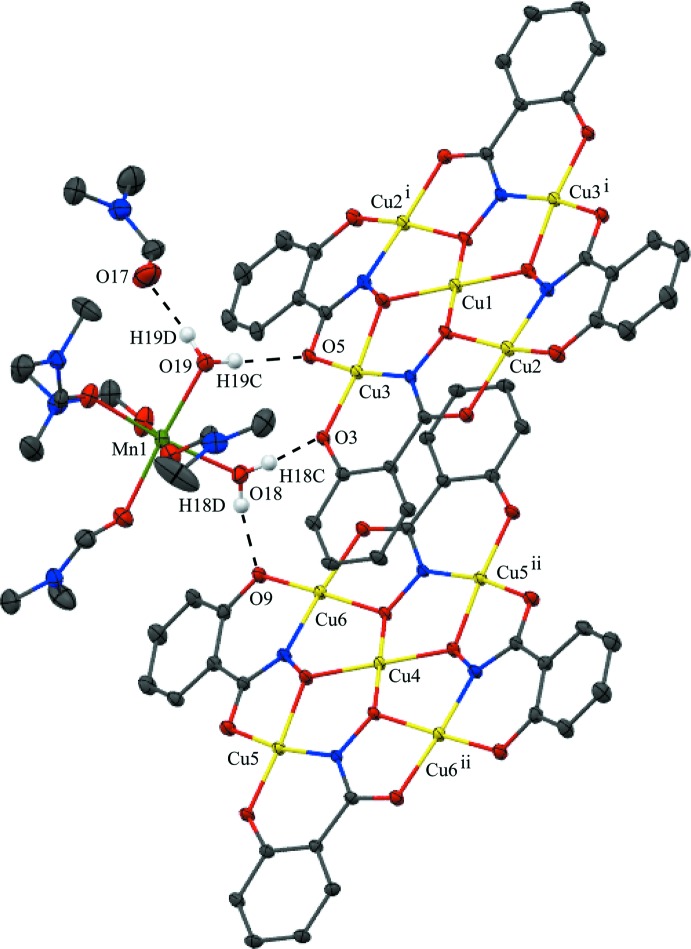
The inter­molecular hydrogen bonds present between neighboring mol­ecules of *cis*-[Mn(H_2_O)_2_(DMF)_4_]{Cu[12-MC_Cu(II)N(shi)_-4]}·DMF with displacement ellipsoids at the 50% probability level [symmetry codes: (i) 2 − *x*, −*y*, 1 − *z* and (ii) 1 − *x*, −*y*, 1 − *z*]. For clarity only the H atoms (white) involved in the hydrogen bonding are displayed. See Fig. 1 ▸ for additional display details.

No strong directional inter­molecular inter­actions are observed between the {Cu[12-MC_Cu(II)N(shi)_-4]}^2−^ anions, but a number of hydrogen bonds exist between the MCs and the counter-cation *cis*-[Mn(H_2_O)_2_(DMF)_4_]^2+^ and between the counter-cation and the lattice DMF mol­ecule (Table 4 ▸, Fig. 3 ▸). The water mol­ecule associated with O18 of the Mn^II^ cation forms hydrogen bonds to both MC anions. The hydrogen bonds are to phenolate oxygen atoms (O18—H18*C*⋯O3 and O18—H18*D*⋯O9) of the neighboring MCs. The water mol­ecule associated with O19 of the Mn^II^ cation forms hydrogen bonds to a carbonyl oxygen atom of the MC associated with Cu1 (O19—H19*C*⋯O5) and to the carbonyl oxygen atom of the lattice DMF mol­ecule (O19—H19*D*⋯O17). These hydrogen-bonding inter­actions, in addition to pure van der Waals forces, contribute to the overall packing of the mol­ecules.

## Database survey   

As stated above, the Cambridge Structural Database (CSD version 5.41, update March 2020; Groom *et al.*, 2016 ▸) lists 35 different penta­copper(II) 12-metallacrown-4 complexes with four ring Cu^II^ ions and one central Cu^II^ ion. A variety of different ligands are used to generate the MCs, but only four structures use the ligand salicyl­hydroximate to build the {Cu^II^[12-MC_Cu(II)_-4]}^2−^ framework. The counter-cations in the four other structures are tetra­methyl­ammonium (YELTOY; Gibney *et al.*, 1994 ▸), [Na(15-crown-5)]^+^ (YELTIS; Gibney *et al.*, 1994 ▸), tetra­ethyl­ammonium (UNOTUN; Herring *et al.*, 2011 ▸), and tri­ethyl­ammonium (COLVAC; Happ & Rentschler, 2014 ▸). For the structures with tetra­methyl­ammonium, tetra­ethyl­ammonium, tri­ethyl­ammonium, and *cis*-[Mn(H_2_O)_2_(DMF)_4_]^2+^, the cations are located in the lattice, and the {Cu[12-MC_Cu(II)N(shi)_-4]}^2−^ anions can be considered nearly planar with a ‘mol­ecular disk’ configuration or slightly to significantly non-planar with a ‘sofa’ configuration. As originally described by Pecoraro and coworkers (Gibney *et al.*, 1994 ▸), in the mol­ecular disk configuration the benzene rings of the shi^3−^ ligands lie approximately in the same plane, and in the sofa configuration two of the benzene rings are tilted upwards relative to the MC central cavity and the two opposite benzene rings are tilted downwards. Lastly, for the structure with [Na(15-crown-5)]^+^, the two cations are bound to the phenolate and carbonyl oxygen atoms of the {Cu[12-MC_Cu(II)N(shi)_-4]}^2−^ anion. This causes the MC to become domed with the benzene rings pointing downwards relative to the MC central cavity and the [Na(15-crown-5)]^+^ cations bonded to the convex side of the MC.

## Synthesis and crystallization   

Manganese(II) chloride tetra­hydrate (Certified ACS) was purchased from Fisher Scientific. Copper(II) chloride dihydrate was purchased from J. T. Baker Chemical Company. Salicyl­hydroxamic acid (99%) was purchased from Alfa Aesar. Tri­ethano­lamine (98%) was purchased from Sigma–Aldrich. *N*,*N*-Di­methyl­formamide (DMF, Certified ACS) was purchased from BDH Chemicals. All reagents were used as received without further purification.

Salicyl­hydroxamic acid (H_3_shi; 0.1541 g, 1 mmol) and copper(II) chloride dihydrate (0.1705 g, 1 mmol) were dissolved in 10 mL of DMF resulting in a green–brown solution. Tri­ethano­lamine (0.3764 g, 2.5 mmol) was then added to the CuCl_2_/H_3_shi solution, resulting in a dark-green color. Separately, manganese(II) chloride tetra­hydrate (0.7891 g, 4 mmol) was dissolved in 20 mL of DMF, resulting in a clear and colorless solution. The MnCl_2_ solution was then added to the CuCl_2_/H_3_shi/tri­ethano­lamine solution and no color change was observed. The solution was stirred overnight and then gravity filtered the next day. A dark-green precipitate was recovered and discarded. The filtrate was a dark-green color. The solution was left for slow evaporation at room temperature, and after 26 days dark-green plate-shaped crystals were collected for X-ray analysis. The remaining crystals were collected, washed with cold DMF, and dried. The percentage yield of the reaction was 57% (0.1576 g, 0.1147 mmol) based on copper(II) chloride dihydrate.

## Refinement   

Two crystallographically independent metallacrown anions are present in the structure. Both are located on crystallographic inversion centers with the central of the five copper atoms situated on the inversion center. Both anions exhibit minor main mol­ecule disorder by an approximate (non-crystallographic) 180° rotation. A *cis*-[Mn(H_2_O)_2_(DMF)_4_]^2+^ cation and a solvate DMF mol­ecule are located in the lattice and are not disordered. The major and minor disordered moieties of both anions were each restrained to have similar geometries (*SHELXL* SAME commands). *U* ^i*j*^ components of ADPs for disordered atoms closer to each other than 2.0 Å were restrained to be similar. Subject to these conditions the occupancy ratio refined to 0.9010 (9) to 0.0990 (9) for the anion associated with Cu1 and 0.9497 (8) to 0.0503 (8) for the anion associated with Cu4. Water hydrogen-atom positions were refined and O—H distances restrained to 0.84 (2) Å. Additional crystallographic data and experimental parameters are provided in Table 5 ▸ and the CIF of the compound.

Hydrogen atoms attached to carbon atoms as well as hydroxyl hydrogen atoms were positioned geometrically and constrained to ride on their parent atoms. Carbon–hydrogen bond distances were constrained to 0.95 Å for aromatic and aldehyde C—H moieties, and to 0.98 Å for CH_3_ moieties. Oxygen–hydrogen distances of alcohols were constrained to 0.84 Å and were allowed to rotate but not to tip to best fit the experimental electron density. *U*_iso_(H) values were set to *kU*_eq_(C,O) with *k* = 1.5 for CH_3_ and OH, and 1.2 for C—H units.

## Supplementary Material

Crystal structure: contains datablock(s) I, global. DOI: 10.1107/S2056989020005770/yk2128sup1.cif

Structure factors: contains datablock(s) I. DOI: 10.1107/S2056989020005770/yk2128Isup2.hkl

CCDC reference: 1999315

Additional supporting information:  crystallographic information; 3D view; checkCIF report

## Figures and Tables

**Figure 2:**
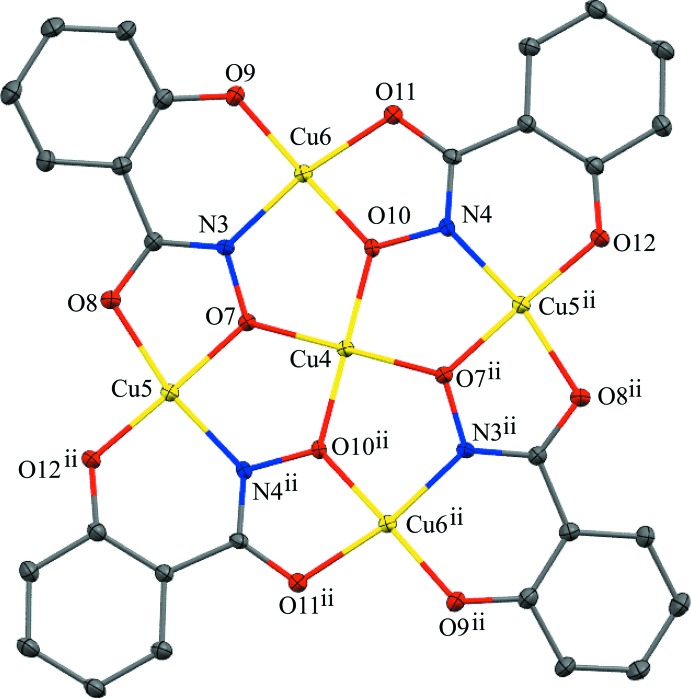
The single-crystal X-ray structure of {Cu[12-MC_Cu(II)N(shi)_-4]}^2−^ associated with Cu4 with displacement ellipsoids at the 50% probability level [symmetry code: (ii) 1 − *x*, −*y*, 1 − *z*]. For clarity, only non-carbon atoms have been labeled, and the MC associated with Cu1, the Mn^II^ counter-cation, the lattice DMF mol­ecule, H atoms, and disorder have been omitted. See Fig. 1 ▸ for additional display details.

**Table 1:** Average bond-length (Å) and bond-valence-sum (BVS; v.u.) values used to support the assigned oxidation states of the copper and manganese ions

	Avg. bond length	BVS value	Assigned oxidation state
Mn1	2.171	2.01	2+
Cu1	1.896	2.05	2+
Cu2	1.914	2.11	2+
Cu3	1.921	2.08	2+
Cu4	1.892	2.08	2+
Cu5	1.922	2.07	2+
Cu6	1.912	2.12	2+

**Table 2:** Continuous shape measurement (CShM) values (*SHAPE 2.1*; Llunell *et al.*, 2013 ▸) for the four-coordinate copper(II) ions

	Square	Tetra­hedron	Seesaw	Vacant trigonal bipyramid
Cu1	0.025	33.350	19.048	34.881
Cu2	1.870	22.459	11.479	23.026
Cu3	0.404	30.267	16.579	30.405
Cu4	0.027	33.352	19.058	34.887
Cu5	0.435	30.734	16.919	30.682
Cu6	0.606	28.098	15.366	28.580

**Table 3:** Continuous shape measurement (CShM) values (*SHAPE 2.1*; Llunell *et al.*, 2013 ▸) for the six-coordinate manganese(II) ion

	Hexagon	Penta­gonal pyramid	Octa­hedron	Trigonal prism	Johnson pentagonal pyramid
Mn1	32.455	27.045	0.240	14.096	30.823

**Table 4:** Hydrogen-bond geometry (Å, °)

*D*—H⋯*A*	*D*—H	H⋯*A*	*D*⋯*A*	*D*—H⋯*A*
O18—H18*C*⋯O3	0.83 (2)	2.07 (3)	2.847 (3)	156 (5)
O18—H18*D*⋯O9	0.84 (2)	1.95 (2)	2.778 (3)	169 (5)
O19—H19*C*⋯O5	0.83 (2)	1.93 (2)	2.746 (3)	167 (5)
O19—H19*D*⋯O17	0.84 (2)	1.88 (2)	2.713 (4)	175 (5)

**Table 5:** Experimental details

Crystal data	
Chemical formula	[Mn(C_3_H_7_NO)_4_(H_2_O)_2_][Cu_5_(C_7_H_4_NO_3_)_4_]·C_3_H_7_NO
*M*_r_	1374.60
Crystal system, space group	Monoclinic, *P*2_1_/*n*
Temperature (K)	100
*a*, *b*, *c* (Å)	19.0669 (9), 14.2943 (6), 19.3450 (8)
β (°)	95.1476 (18)
*V* (Å^3^)	5251.2 (4)
*Z*	4
Radiation type	Mo *K*α
μ (mm^−1^)	2.30
Crystal size (mm)	0.45 × 0.41 × 0.25

Data collection	
Diffractometer	Bruker AXS D8 Quest CMOS diffractometer
Absorption correction	Multi-scan (*SADABS2016/2*; Krause *et al.*, 2015 ▸)
*T*_min_, *T*_max_	0.582, 0.748
No. of measured, independent and observed [*I* > 2σ(*I*)] reflections	87027, 28700, 18767
*R*_int_	0.043
(sin θ/λ)_max_ (Å^−1^)	0.879

Refinement	
*R*[*F* ^2^ > 2σ(*F* ^2^)], *wR*(*F* ^2^), *S*	0.065, 0.143, 1.07
No. of reflections	28700
No. of parameters	1153
No. of restraints	1810
H-atom treatment	H atoms treated by a mixture of independent and constrained refinement
Δρ_max_, Δρ_min_ (e Å^−3^)	2.44, −2.18

Computer programs: *APEX3* and *SAINT*(Bruker, 2016 ▸), *SHELXS97* (Sheldrick, 2008 ▸), *SHELXL2018/1* (Sheldrick, 2015 ▸), *SHELXLE Rev859* (Hübschle *et al.*, 2011 ▸), *Mercury* (Macrae *et al.*, 2020 ▸) and *publCIF* (Westrip, 2010 ▸).

## supplementary crystallographic information

### Crystal data

**Table:**

[Mn(C_3_H_7_NO)_4_(H_2_O)_2_][Cu_5_(C_7_H_4_NO_3_)_4_]·C_3_H_7_NO	*F*(000) = 2792
*M**_r_* = 1374.60	*D*_x_ = 1.739 Mg m^−^^3^
Monoclinic, *P*2_1_/*n*	Mo *K*α radiation, λ = 0.71073 Å
*a* = 19.0669 (9) Å	Cell parameters from 9700 reflections
*b* = 14.2943 (6) Å	θ = 2.8–38.6°
*c* = 19.3450 (8) Å	µ = 2.30 mm^−^^1^
β = 95.1476 (18)°	*T* = 100 K
*V* = 5251.2 (4) Å^3^	Block, dark green
*Z* = 4	0.45 × 0.41 × 0.25 mm

### Data collection

**Table:**

Bruker AXS D8 Quest CMOS diffractometer	28700 independent reflections
Radiation source: sealed tube X-ray source	18767 reflections with *I* > 2σ(*I*)
Triumph curved graphite crystal monochromator	*R*_int_ = 0.043
ω and phi scans	θ_max_ = 38.7°, θ_min_ = 2.7°
Absorption correction: multi-scan (*SADABS2016/2*; Krause *et al.*, 2015)	*h* = −33→33
*T*_min_ = 0.582, *T*_max_ = 0.748	*k* = −25→25
87027 measured reflections	*l* = −33→31

### Refinement

**Table:**

Refinement on *F*^2^	Primary atom site location: structure-invariant direct methods
Least-squares matrix: full	Secondary atom site location: difference Fourier map
*R*[*F*^2^ > 2σ(*F*^2^)] = 0.065	Hydrogen site location: mixed
*wR*(*F*^2^) = 0.143	H atoms treated by a mixture of independent and constrained refinement
*S* = 1.07	*w* = 1/[σ^2^(*F*_o_^2^) + (0.0001*P*)^2^ + 29.7569*P*] where *P* = (*F*_o_^2^ + 2*F*_c_^2^)/3
28700 reflections	(Δ/σ)_max_ = 0.001
1153 parameters	Δρ_max_ = 2.44 e Å^−^^3^
1810 restraints	Δρ_min_ = −2.18 e Å^−^^3^

### Special details

**Table:**

Geometry. All esds (except the esd in the dihedral angle between two l.s. planes) are estimated using the full covariance matrix. The cell esds are taken into account individually in the estimation of esds in distances, angles and torsion angles; correlations between esds in cell parameters are only used when they are defined by crystal symmetry. An approximate (isotropic) treatment of cell esds is used for estimating esds involving l.s. planes.
Refinement. Two crystallographically independent metallacrown anions are present in the structure. Both are located on crystallographic inversion centers, with the central of the five copper atoms situated on the inversion center. Both anions exhibit minor main molecule disorder, by an approximate (non- crystallographic) 180 degree rotation. Not disordered are the Mn(DMF)4(H2O)2 cation and a solvate DMF molecule. The major and minor disordered moieties of both anions were each restrained to have similar geometries. Uij components of ADPs for disordered atoms closer to each other than 1.7 Angstrom were restrained to be similar. Subject to these conditions the occupancy ratio refined to 0.9010 (9) to 0.0990 (9) for the first molecule and 0.9497 (8) to 0.0503 (8) for the second. Water H atom positions were refined and O-H distances restrained to 0.84 (2) Angstrom.

### Fractional atomic coordinates and isotropic or equivalent isotropic displacement parameters (Å^2^)

**Table:**

	*x*	*y*	*z*	*U*_iso_*/*U*_eq_	Occ. (<1)
Cu1	1.000000	0.000000	0.500000	0.01188 (8)	
Cu2	0.93260 (2)	−0.11523 (3)	0.62324 (2)	0.01279 (7)	0.9010 (9)
O1	0.93764 (10)	−0.00304 (15)	0.57089 (11)	0.0129 (3)	0.9010 (9)
N1	0.87242 (17)	0.0428 (3)	0.5582 (3)	0.0111 (6)	0.9010 (9)
O2	0.83769 (11)	−0.06858 (15)	0.63222 (11)	0.0139 (3)	0.9010 (9)
C1	0.82431 (14)	0.00355 (19)	0.59223 (14)	0.0116 (4)	0.9010 (9)
C2	0.75232 (14)	0.0439 (2)	0.58686 (15)	0.0119 (4)	0.9010 (9)
C3	0.70219 (15)	−0.0011 (2)	0.62449 (15)	0.0154 (5)	0.9010 (9)
H3	0.715606	−0.055972	0.649998	0.019*	0.9010 (9)
C4	0.6337 (2)	0.0320 (3)	0.6257 (2)	0.0175 (7)	0.9010 (9)
H4	0.600872	0.000413	0.651515	0.021*	0.9010 (9)
C5	0.61435 (18)	0.1129 (3)	0.5881 (2)	0.0163 (6)	0.9010 (9)
H5	0.568246	0.137661	0.589364	0.020*	0.9010 (9)
C6	0.66184 (15)	0.1572 (2)	0.54889 (15)	0.0142 (4)	0.9010 (9)
H6	0.647143	0.211457	0.523118	0.017*	0.9010 (9)
C7	0.73162 (14)	0.12434 (19)	0.54601 (14)	0.0121 (4)	0.9010 (9)
O3	0.77261 (11)	0.17166 (15)	0.50602 (11)	0.0136 (3)	0.9010 (9)
Cu3	0.86525 (2)	0.13811 (2)	0.48811 (2)	0.01182 (7)	0.9010 (9)
O4	0.96260 (10)	0.11912 (15)	0.47381 (11)	0.0135 (3)	0.9010 (9)
N2	0.98210 (16)	0.1601 (2)	0.41233 (15)	0.0136 (6)	0.9010 (9)
O5	0.87361 (11)	0.22739 (15)	0.41292 (11)	0.0137 (3)	0.9010 (9)
C8	0.93312 (14)	0.21663 (19)	0.38474 (14)	0.0125 (4)	0.9010 (9)
C9	0.94586 (16)	0.2689 (2)	0.32167 (15)	0.0132 (4)	0.9010 (9)
C10	0.89277 (17)	0.3304 (2)	0.29445 (15)	0.0168 (5)	0.9010 (9)
H10	0.850788	0.335727	0.317159	0.020*	0.9010 (9)
C11	0.8998 (2)	0.3832 (3)	0.2359 (2)	0.0210 (7)	0.9010 (9)
H11	0.862623	0.423050	0.217913	0.025*	0.9010 (9)
C12	0.9626 (3)	0.3775 (4)	0.2031 (3)	0.0236 (8)	0.9010 (9)
H12	0.968674	0.414714	0.163392	0.028*	0.9010 (9)
C13	1.01514 (18)	0.3176 (2)	0.22884 (18)	0.0221 (6)	0.9010 (9)
H13	1.057372	0.314608	0.206328	0.027*	0.9010 (9)
C14	1.00866 (16)	0.2603 (2)	0.28744 (15)	0.0161 (5)	0.9010 (9)
O6	1.06184 (12)	0.20319 (17)	0.30490 (12)	0.0185 (4)	0.9010 (9)
Cu4	0.500000	0.000000	0.500000	0.01170 (8)	
Cu5	0.36653 (2)	0.14212 (2)	0.49259 (2)	0.01211 (6)	0.9497 (8)
O7	0.46380 (10)	0.12115 (14)	0.47964 (11)	0.0145 (3)	0.9497 (8)
N3	0.48669 (13)	0.1680 (2)	0.42164 (17)	0.0141 (5)	0.9497 (8)
O8	0.37971 (11)	0.23815 (15)	0.42153 (11)	0.0171 (4)	0.9497 (8)
C15	0.44041 (13)	0.22865 (18)	0.39663 (14)	0.0126 (4)	0.9497 (8)
C16	0.45885 (14)	0.28759 (19)	0.33773 (14)	0.0138 (4)	0.9497 (8)
C17	0.40423 (15)	0.3424 (2)	0.30463 (15)	0.0166 (5)	0.9497 (8)
H17	0.359149	0.341905	0.321939	0.020*	0.9497 (8)
C18	0.41498 (19)	0.3970 (2)	0.2474 (2)	0.0217 (7)	0.9497 (8)
H18	0.377609	0.432908	0.225074	0.026*	0.9497 (8)
C19	0.4820 (2)	0.3984 (5)	0.2231 (2)	0.0221 (7)	0.9497 (8)
H19	0.489739	0.434754	0.183429	0.026*	0.9497 (8)
C20	0.53676 (16)	0.3479 (2)	0.25591 (16)	0.0192 (5)	0.9497 (8)
H20	0.582196	0.352367	0.239636	0.023*	0.9497 (8)
C21	0.52693 (15)	0.28992 (19)	0.31299 (15)	0.0154 (4)	0.9497 (8)
O9	0.58291 (11)	0.24093 (15)	0.33919 (11)	0.0167 (4)	0.9497 (8)
Cu6	0.57576 (2)	0.12989 (2)	0.39086 (2)	0.01213 (6)	0.9497 (8)
O10	0.56539 (10)	0.01070 (13)	0.43250 (10)	0.0125 (3)	0.9497 (8)
N4	0.62916 (14)	−0.0390 (2)	0.4431 (3)	0.0115 (4)	0.9497 (8)
O11	0.66753 (10)	0.07989 (14)	0.37717 (11)	0.0144 (3)	0.9497 (8)
C22	0.67889 (13)	0.00222 (18)	0.41166 (13)	0.0115 (4)	0.9497 (8)
C23	0.74913 (13)	−0.04172 (19)	0.41456 (14)	0.0122 (4)	0.9497 (8)
C24	0.79951 (14)	0.0024 (2)	0.37672 (15)	0.0145 (4)	0.9497 (8)
H24	0.786818	0.058142	0.351927	0.017*	0.9497 (8)
C25	0.86714 (15)	−0.0328 (3)	0.37443 (19)	0.0166 (5)	0.9497 (8)
H25	0.900091	−0.002402	0.348016	0.020*	0.9497 (8)
C26	0.88563 (15)	−0.1144 (2)	0.41205 (17)	0.0173 (5)	0.9497 (8)
H26	0.931656	−0.139508	0.411162	0.021*	0.9497 (8)
C27	0.83747 (14)	−0.1586 (2)	0.45048 (15)	0.0159 (5)	0.9497 (8)
H27	0.851464	−0.213495	0.475762	0.019*	0.9497 (8)
C28	0.76769 (13)	−0.12442 (19)	0.45326 (14)	0.0136 (4)	0.9497 (8)
O12	0.72610 (10)	−0.17222 (15)	0.49136 (11)	0.0159 (4)	0.9497 (8)
Cu2B	1.11148 (18)	0.0603 (2)	0.38816 (17)	0.0151 (7)	0.0990 (9)
O1B	1.0274 (8)	0.0792 (14)	0.4321 (9)	0.018 (2)	0.0990 (9)
N1B	0.9811 (12)	0.146 (2)	0.4009 (14)	0.014 (2)	0.0990 (9)
O2B	1.0686 (8)	0.1604 (13)	0.3283 (9)	0.016 (2)	0.0990 (9)
C1B	1.0078 (9)	0.1843 (15)	0.3481 (11)	0.0136 (19)	0.0990 (9)
C2B	0.9674 (11)	0.2610 (18)	0.3106 (13)	0.016 (2)	0.0990 (9)
C3B	0.9973 (12)	0.3032 (18)	0.2545 (12)	0.018 (2)	0.0990 (9)
H3B	1.044539	0.289257	0.246045	0.022*	0.0990 (9)
C4B	0.958 (2)	0.366 (4)	0.211 (2)	0.022 (3)	0.0990 (9)
H4B	0.973348	0.382693	0.167463	0.027*	0.0990 (9)
C5B	0.895 (2)	0.403 (3)	0.232 (2)	0.020 (3)	0.0990 (9)
H5B	0.872475	0.453475	0.207068	0.024*	0.0990 (9)
C6B	0.8666 (13)	0.3642 (17)	0.2883 (12)	0.018 (3)	0.0990 (9)
H6B	0.823361	0.388191	0.301576	0.021*	0.0990 (9)
C7B	0.8996 (10)	0.2904 (16)	0.3273 (11)	0.016 (2)	0.0990 (9)
O3B	0.8645 (9)	0.2589 (12)	0.3807 (8)	0.015 (2)	0.0990 (9)
Cu3B	0.89646 (17)	0.1672 (2)	0.44622 (18)	0.0156 (6)	0.0990 (9)
O4B	0.9298 (8)	0.0870 (13)	0.5213 (8)	0.016 (2)	0.0990 (9)
N2B	0.8738 (14)	0.055 (4)	0.558 (3)	0.014 (2)	0.0990 (9)
O5B	0.8101 (7)	0.1663 (12)	0.4959 (8)	0.012 (2)	0.0990 (9)
C8B	0.8145 (9)	0.0988 (15)	0.5408 (11)	0.0126 (18)	0.0990 (9)
C9B	0.7503 (9)	0.0747 (16)	0.5741 (12)	0.0117 (19)	0.0990 (9)
C10B	0.6913 (9)	0.1315 (15)	0.5565 (12)	0.013 (2)	0.0990 (9)
H10B	0.694945	0.184109	0.527031	0.015*	0.0990 (9)
C11B	0.6279 (13)	0.110 (3)	0.582 (2)	0.016 (3)	0.0990 (9)
H11B	0.586440	0.143106	0.565392	0.019*	0.0990 (9)
C12B	0.6243 (16)	0.040 (3)	0.633 (2)	0.017 (3)	0.0990 (9)
H12B	0.582932	0.032875	0.656677	0.020*	0.0990 (9)
C13B	0.6816 (10)	−0.0164 (18)	0.6486 (15)	0.020 (3)	0.0990 (9)
H13B	0.676828	−0.068618	0.678149	0.023*	0.0990 (9)
C14B	0.7477 (9)	−0.0004 (15)	0.6220 (12)	0.015 (2)	0.0990 (9)
O6B	0.8013 (9)	−0.0544 (13)	0.6467 (10)	0.017 (2)	0.0990 (9)
Cu5B	0.5982 (4)	−0.1776 (5)	0.5491 (4)	0.0181 (14)	0.0503 (8)
O7B	0.5715 (11)	−0.0883 (18)	0.4778 (15)	0.012 (3)	0.0503 (8)
N3B	0.630 (2)	−0.050 (5)	0.448 (5)	0.013 (2)	0.0503 (8)
O8B	0.6873 (12)	−0.173 (2)	0.5041 (18)	0.017 (3)	0.0503 (8)
C15B	0.6861 (13)	−0.100 (2)	0.464 (2)	0.014 (2)	0.0503 (8)
C16B	0.7516 (13)	−0.075 (2)	0.433 (2)	0.012 (2)	0.0503 (8)
C17B	0.8066 (16)	−0.140 (3)	0.447 (2)	0.014 (2)	0.0503 (8)
H17B	0.798781	−0.195059	0.472320	0.017*	0.0503 (8)
C18B	0.8721 (18)	−0.124 (3)	0.423 (3)	0.016 (3)	0.0503 (8)
H18B	0.909963	−0.166878	0.433122	0.019*	0.0503 (8)
C19B	0.8803 (18)	−0.043 (4)	0.384 (4)	0.016 (3)	0.0503 (8)
H19B	0.925534	−0.028177	0.370588	0.019*	0.0503 (8)
C20B	0.8249 (14)	0.016 (3)	0.365 (2)	0.014 (3)	0.0503 (8)
H20B	0.832841	0.068786	0.337602	0.017*	0.0503 (8)
C21B	0.7566 (13)	0.000 (2)	0.386 (2)	0.013 (2)	0.0503 (8)
O9B	0.7079 (10)	0.065 (2)	0.3686 (18)	0.015 (2)	0.0503 (8)
Cu6B	0.6171 (3)	0.0670 (4)	0.3977 (3)	0.0140 (11)	0.0503 (8)
O10B	0.5253 (11)	0.0771 (18)	0.4283 (18)	0.014 (2)	0.0503 (8)
N4B	0.499 (2)	0.169 (3)	0.422 (3)	0.013 (2)	0.0503 (8)
O11B	0.5872 (13)	0.1851 (17)	0.3533 (17)	0.014 (3)	0.0503 (8)
C22B	0.5238 (15)	0.206 (2)	0.366 (2)	0.013 (2)	0.0503 (8)
C23B	0.4866 (17)	0.285 (3)	0.330 (2)	0.015 (2)	0.0503 (8)
C24B	0.522 (2)	0.330 (3)	0.279 (2)	0.017 (2)	0.0503 (8)
H24B	0.569923	0.316021	0.275208	0.020*	0.0503 (8)
C25B	0.488 (4)	0.394 (9)	0.234 (5)	0.020 (3)	0.0503 (8)
H25B	0.509485	0.418379	0.195293	0.024*	0.0503 (8)
C26B	0.419 (3)	0.422 (5)	0.247 (3)	0.021 (3)	0.0503 (8)
H26B	0.394347	0.465332	0.216809	0.025*	0.0503 (8)
C27B	0.387 (2)	0.387 (3)	0.303 (2)	0.021 (3)	0.0503 (8)
H27B	0.344128	0.413036	0.314445	0.025*	0.0503 (8)
C28B	0.4181 (17)	0.313 (3)	0.345 (2)	0.017 (2)	0.0503 (8)
O12B	0.3842 (16)	0.286 (3)	0.3987 (17)	0.016 (3)	0.0503 (8)
C29	0.58473 (18)	0.5419 (2)	0.45772 (18)	0.0263 (6)	
H29	0.598215	0.556341	0.504979	0.032*	
C30	0.5058 (2)	0.5667 (4)	0.3558 (2)	0.0458 (11)	
H30A	0.499783	0.626894	0.331718	0.069*	
H30B	0.461097	0.532462	0.351376	0.069*	
H30C	0.541908	0.529930	0.335133	0.069*	
C31	0.4857 (2)	0.6472 (3)	0.4664 (3)	0.0419 (10)	
H31A	0.493158	0.711397	0.450845	0.063*	
H31B	0.500490	0.642121	0.516080	0.063*	
H31C	0.435621	0.631332	0.458142	0.063*	
C32	0.74497 (18)	0.3618 (2)	0.58878 (16)	0.0227 (5)	
H32	0.777172	0.323690	0.566730	0.027*	
C33	0.6892 (3)	0.3988 (6)	0.6927 (2)	0.075 (2)	
H33A	0.665826	0.445893	0.661825	0.112*	
H33B	0.714479	0.429880	0.732630	0.112*	
H33C	0.653897	0.356164	0.708838	0.112*	
C34	0.7762 (3)	0.2693 (3)	0.6914 (2)	0.0387 (9)	
H34A	0.742553	0.220454	0.701746	0.058*	
H34B	0.800563	0.292273	0.734884	0.058*	
H34C	0.810620	0.243354	0.662021	0.058*	
C35	0.75700 (17)	0.6276 (2)	0.53074 (16)	0.0208 (5)	
H35	0.710755	0.650430	0.534728	0.025*	
C36	0.8771 (2)	0.6121 (4)	0.5799 (2)	0.0456 (11)	
H36A	0.881711	0.579999	0.535730	0.068*	
H36B	0.888563	0.568592	0.618356	0.068*	
H36C	0.909573	0.665410	0.584156	0.068*	
C37	0.78793 (19)	0.6978 (3)	0.64266 (19)	0.0305 (7)	
H37A	0.821025	0.749865	0.650914	0.046*	
H37B	0.791271	0.656399	0.683221	0.046*	
H37C	0.739874	0.722243	0.634735	0.046*	
C38	0.7762 (2)	0.5562 (3)	0.30981 (18)	0.0320 (7)	
H38	0.818999	0.544599	0.289661	0.038*	
C39	0.6864 (2)	0.6694 (3)	0.3355 (3)	0.0426 (10)	
H39A	0.652816	0.696108	0.299528	0.064*	
H39B	0.697726	0.715999	0.371940	0.064*	
H39C	0.665538	0.614152	0.355611	0.064*	
C40	0.7863 (3)	0.7156 (3)	0.2692 (2)	0.0411 (10)	
H40A	0.830634	0.691374	0.254269	0.062*	
H40B	0.796246	0.768504	0.300784	0.062*	
H40C	0.756005	0.736452	0.228507	0.062*	
C41	0.9701 (2)	0.5292 (3)	0.3821 (2)	0.0357 (8)	
H41	0.966755	0.476397	0.352148	0.043*	
C42	1.0251 (3)	0.6804 (3)	0.4111 (3)	0.0452 (10)	
H42A	1.072249	0.676404	0.435695	0.068*	
H42B	0.989763	0.681872	0.444855	0.068*	
H42C	1.021508	0.737459	0.382967	0.068*	
C43	1.0527 (3)	0.5938 (4)	0.3057 (3)	0.0506 (12)	
H43A	1.037418	0.538532	0.278324	0.076*	
H43B	1.103051	0.588642	0.320481	0.076*	
H43C	1.044206	0.650101	0.277271	0.076*	
N5	0.52711 (15)	0.5827 (2)	0.42772 (16)	0.0258 (5)	
N6	0.73860 (16)	0.3463 (2)	0.65531 (14)	0.0285 (6)	
N7	0.80522 (14)	0.64533 (19)	0.58180 (15)	0.0231 (5)	
N8	0.75091 (17)	0.6422 (2)	0.30472 (16)	0.0283 (6)	
N9	1.01309 (17)	0.5998 (2)	0.36654 (19)	0.0338 (7)	
O13	0.62166 (13)	0.48689 (18)	0.42845 (15)	0.0299 (5)	
O14	0.71231 (13)	0.42205 (18)	0.55295 (12)	0.0248 (4)	
O15	0.76761 (14)	0.58292 (17)	0.47750 (13)	0.0265 (5)	
O16	0.74914 (17)	0.48880 (18)	0.33857 (13)	0.0333 (6)	
O17	0.93532 (18)	0.5286 (2)	0.4319 (2)	0.0508 (9)	
O18	0.70585 (12)	0.30438 (16)	0.41214 (13)	0.0232 (4)	
H18C	0.728 (2)	0.259 (2)	0.428 (2)	0.035*	
H18D	0.6671 (15)	0.293 (3)	0.390 (2)	0.035*	
O19	0.83912 (12)	0.39926 (16)	0.46351 (13)	0.0218 (4)	
H19C	0.849 (2)	0.351 (2)	0.442 (2)	0.033*	
H19D	0.8702 (19)	0.439 (3)	0.456 (2)	0.033*	
Mn1	0.73098 (2)	0.44759 (3)	0.44234 (2)	0.01756 (8)	

### Atomic displacement parameters (Å^2^)

**Table:**

	*U*^11^	*U*^22^	*U*^33^	*U*^12^	*U*^13^	*U*^23^
Cu1	0.01194 (17)	0.01244 (17)	0.01186 (17)	0.00303 (14)	0.00427 (13)	0.00407 (14)
Cu2	0.01307 (14)	0.01378 (14)	0.01206 (14)	0.00336 (11)	0.00406 (11)	0.00473 (11)
O1	0.0107 (7)	0.0147 (8)	0.0136 (8)	0.0052 (6)	0.0022 (6)	0.0056 (7)
N1	0.0111 (9)	0.0109 (15)	0.0117 (9)	0.0024 (8)	0.0035 (8)	0.0028 (11)
O2	0.0136 (8)	0.0142 (8)	0.0145 (9)	0.0019 (7)	0.0043 (7)	0.0042 (7)
C1	0.0112 (9)	0.0130 (10)	0.0111 (9)	0.0020 (8)	0.0044 (8)	0.0006 (8)
C2	0.0098 (9)	0.0143 (11)	0.0118 (10)	0.0023 (8)	0.0020 (8)	0.0006 (9)
C3	0.0147 (11)	0.0166 (11)	0.0155 (11)	0.0009 (9)	0.0039 (9)	0.0036 (9)
C4	0.0133 (13)	0.0223 (15)	0.0177 (15)	0.0033 (10)	0.0059 (10)	0.0021 (12)
C5	0.0105 (12)	0.0219 (13)	0.0170 (13)	0.0017 (11)	0.0046 (10)	−0.0007 (11)
C6	0.0124 (10)	0.0169 (11)	0.0133 (11)	0.0028 (9)	0.0015 (8)	0.0008 (9)
C7	0.0116 (9)	0.0131 (10)	0.0117 (10)	0.0007 (8)	0.0022 (8)	−0.0013 (8)
O3	0.0124 (8)	0.0133 (8)	0.0156 (9)	0.0028 (6)	0.0048 (7)	0.0028 (7)
Cu3	0.01186 (13)	0.01167 (13)	0.01247 (14)	0.00279 (11)	0.00411 (11)	0.00333 (11)
O4	0.0131 (8)	0.0153 (8)	0.0132 (8)	0.0042 (6)	0.0065 (6)	0.0065 (7)
N2	0.0132 (9)	0.0143 (12)	0.0138 (12)	0.0026 (8)	0.0045 (8)	0.0041 (9)
O5	0.0136 (8)	0.0125 (8)	0.0156 (9)	0.0033 (6)	0.0042 (7)	0.0042 (7)
C8	0.0128 (10)	0.0123 (10)	0.0127 (10)	0.0026 (8)	0.0024 (8)	0.0019 (8)
C9	0.0154 (11)	0.0124 (10)	0.0120 (10)	0.0012 (9)	0.0024 (8)	0.0034 (8)
C10	0.0214 (12)	0.0158 (11)	0.0134 (11)	0.0037 (10)	0.0024 (9)	0.0019 (9)
C11	0.0271 (15)	0.0201 (18)	0.0156 (13)	0.0083 (13)	0.0018 (11)	0.0068 (12)
C12	0.0304 (16)	0.024 (2)	0.0168 (16)	0.0056 (15)	0.0060 (12)	0.0094 (12)
C13	0.0239 (14)	0.0229 (14)	0.0201 (13)	0.0037 (11)	0.0054 (11)	0.0089 (11)
C14	0.0177 (11)	0.0159 (11)	0.0147 (11)	0.0021 (9)	0.0018 (9)	0.0058 (9)
O6	0.0169 (9)	0.0208 (10)	0.0180 (10)	0.0052 (8)	0.0037 (7)	0.0114 (8)
Cu4	0.01144 (16)	0.01056 (16)	0.01369 (18)	0.00205 (13)	0.00444 (14)	0.00307 (14)
Cu5	0.01072 (12)	0.01202 (13)	0.01408 (14)	0.00163 (10)	0.00377 (10)	0.00281 (11)
O7	0.0128 (7)	0.0136 (8)	0.0182 (9)	0.0037 (6)	0.0063 (6)	0.0064 (7)
N3	0.0106 (10)	0.0137 (9)	0.0190 (10)	0.0021 (8)	0.0063 (9)	0.0061 (8)
O8	0.0137 (8)	0.0176 (9)	0.0208 (9)	0.0029 (7)	0.0065 (7)	0.0077 (8)
C15	0.0121 (9)	0.0124 (9)	0.0135 (10)	0.0011 (7)	0.0024 (8)	0.0024 (8)
C16	0.0142 (10)	0.0146 (10)	0.0129 (10)	0.0010 (8)	0.0031 (8)	0.0027 (8)
C17	0.0140 (10)	0.0193 (11)	0.0167 (11)	0.0034 (9)	0.0018 (9)	0.0051 (9)
C18	0.0217 (13)	0.0238 (15)	0.0195 (12)	0.0049 (12)	0.0012 (10)	0.0100 (12)
C19	0.0242 (14)	0.0262 (15)	0.0166 (18)	0.0055 (12)	0.0062 (13)	0.0101 (13)
C20	0.0201 (12)	0.0199 (12)	0.0183 (12)	0.0059 (9)	0.0061 (10)	0.0098 (10)
C21	0.0176 (11)	0.0131 (10)	0.0158 (11)	0.0009 (8)	0.0027 (9)	0.0031 (8)
O9	0.0144 (8)	0.0153 (8)	0.0205 (9)	0.0027 (7)	0.0029 (7)	0.0077 (7)
Cu6	0.01220 (13)	0.01163 (13)	0.01306 (13)	0.00194 (10)	0.00392 (10)	0.00294 (11)
O10	0.0103 (7)	0.0125 (7)	0.0152 (8)	0.0043 (6)	0.0035 (6)	0.0042 (6)
N4	0.0092 (8)	0.0110 (11)	0.0148 (11)	0.0026 (7)	0.0034 (7)	0.0019 (8)
O11	0.0133 (8)	0.0147 (8)	0.0158 (8)	0.0029 (6)	0.0044 (6)	0.0037 (7)
C22	0.0107 (9)	0.0139 (9)	0.0103 (9)	0.0011 (7)	0.0037 (7)	0.0001 (8)
C23	0.0108 (9)	0.0138 (10)	0.0121 (10)	0.0011 (7)	0.0024 (7)	0.0007 (8)
C24	0.0128 (10)	0.0151 (10)	0.0162 (11)	−0.0004 (8)	0.0042 (8)	0.0011 (9)
C25	0.0122 (11)	0.0193 (12)	0.0191 (15)	0.0000 (10)	0.0058 (11)	−0.0001 (10)
C26	0.0115 (10)	0.0206 (12)	0.0206 (13)	0.0003 (9)	0.0055 (9)	0.0002 (10)
C27	0.0124 (10)	0.0183 (11)	0.0174 (11)	0.0035 (8)	0.0030 (9)	0.0017 (9)
C28	0.0118 (9)	0.0144 (10)	0.0150 (10)	0.0010 (8)	0.0037 (8)	−0.0003 (8)
O12	0.0110 (7)	0.0162 (8)	0.0213 (9)	0.0030 (6)	0.0061 (7)	0.0058 (7)
Cu2B	0.0154 (13)	0.0172 (14)	0.0129 (13)	0.0043 (11)	0.0018 (10)	0.0038 (11)
O1B	0.018 (4)	0.022 (4)	0.016 (4)	0.003 (4)	0.010 (4)	0.008 (4)
N1B	0.015 (4)	0.014 (4)	0.014 (4)	0.002 (4)	0.004 (4)	0.008 (4)
O2B	0.014 (4)	0.020 (4)	0.015 (4)	0.004 (4)	0.003 (4)	0.007 (4)
C1B	0.013 (3)	0.014 (3)	0.014 (3)	0.003 (3)	0.004 (3)	0.006 (3)
C2B	0.018 (4)	0.016 (4)	0.015 (4)	0.004 (4)	0.001 (3)	0.004 (3)
C3B	0.021 (4)	0.019 (4)	0.014 (4)	0.000 (4)	0.006 (4)	0.008 (4)
C4B	0.029 (5)	0.021 (5)	0.016 (5)	0.003 (5)	0.002 (4)	0.008 (4)
C5B	0.025 (5)	0.019 (5)	0.015 (5)	0.005 (5)	0.001 (4)	0.006 (4)
C6B	0.020 (5)	0.018 (5)	0.015 (5)	0.011 (5)	−0.002 (5)	0.002 (5)
C7B	0.016 (4)	0.015 (4)	0.016 (4)	0.006 (4)	−0.001 (4)	0.001 (4)
O3B	0.015 (4)	0.015 (4)	0.014 (4)	0.003 (4)	0.004 (4)	0.005 (4)
Cu3B	0.0142 (12)	0.0160 (13)	0.0174 (14)	0.0048 (10)	0.0053 (10)	0.0035 (11)
O4B	0.013 (4)	0.017 (4)	0.019 (4)	0.006 (3)	0.002 (3)	0.003 (4)
N2B	0.011 (4)	0.015 (4)	0.015 (4)	0.003 (4)	0.002 (4)	0.001 (4)
O5B	0.012 (4)	0.014 (4)	0.013 (4)	0.006 (4)	0.004 (4)	0.001 (4)
C8B	0.012 (3)	0.015 (3)	0.012 (3)	0.003 (3)	0.004 (3)	0.001 (3)
C9B	0.010 (3)	0.013 (4)	0.012 (3)	0.005 (3)	0.003 (3)	0.002 (3)
C10B	0.008 (4)	0.015 (4)	0.014 (4)	0.000 (4)	0.000 (3)	0.002 (4)
C11B	0.012 (5)	0.021 (4)	0.015 (5)	0.002 (4)	0.004 (4)	−0.002 (4)
C12B	0.014 (5)	0.019 (5)	0.018 (5)	0.002 (4)	0.003 (4)	0.000 (5)
C13B	0.018 (5)	0.021 (5)	0.020 (5)	−0.001 (5)	0.004 (5)	0.003 (5)
C14B	0.014 (4)	0.017 (4)	0.013 (4)	0.001 (4)	0.004 (4)	0.002 (4)
O6B	0.017 (4)	0.019 (4)	0.015 (4)	0.000 (4)	−0.001 (4)	0.007 (4)
Cu5B	0.016 (3)	0.018 (3)	0.021 (3)	0.008 (2)	0.008 (2)	0.000 (2)
O7B	0.008 (5)	0.013 (5)	0.016 (5)	0.004 (5)	0.002 (5)	0.001 (5)
N3B	0.010 (4)	0.015 (4)	0.016 (4)	0.003 (4)	0.003 (4)	0.001 (4)
O8B	0.013 (6)	0.016 (6)	0.021 (6)	0.007 (5)	0.001 (6)	0.003 (6)
C15B	0.011 (4)	0.015 (4)	0.016 (4)	0.001 (4)	0.003 (4)	0.001 (4)
C16B	0.010 (4)	0.013 (4)	0.014 (4)	0.002 (4)	0.004 (4)	0.002 (4)
C17B	0.010 (4)	0.015 (4)	0.017 (4)	0.002 (4)	0.003 (4)	0.003 (4)
C18B	0.011 (5)	0.017 (5)	0.018 (5)	0.003 (5)	0.004 (5)	−0.001 (5)
C19B	0.011 (5)	0.018 (5)	0.019 (5)	0.000 (5)	0.004 (5)	0.000 (5)
C20B	0.011 (5)	0.018 (5)	0.015 (5)	−0.002 (5)	0.006 (5)	−0.001 (5)
C21B	0.012 (4)	0.014 (4)	0.014 (4)	−0.002 (4)	0.004 (4)	0.000 (4)
O9B	0.013 (4)	0.016 (4)	0.016 (4)	−0.001 (4)	0.001 (4)	0.003 (4)
Cu6B	0.011 (2)	0.015 (2)	0.017 (2)	0.0027 (17)	0.0020 (17)	0.0016 (18)
O10B	0.013 (4)	0.012 (4)	0.017 (4)	0.003 (4)	0.003 (4)	0.003 (4)
N4B	0.013 (4)	0.012 (4)	0.015 (4)	0.002 (4)	0.004 (4)	0.006 (4)
O11B	0.013 (5)	0.011 (5)	0.017 (5)	−0.002 (5)	0.005 (5)	0.004 (5)
C22B	0.013 (3)	0.012 (3)	0.014 (3)	0.001 (3)	0.005 (3)	0.005 (3)
C23B	0.015 (4)	0.015 (4)	0.015 (4)	0.001 (4)	0.003 (4)	0.005 (4)
C24B	0.018 (4)	0.019 (4)	0.014 (4)	0.001 (4)	0.007 (4)	0.006 (4)
C25B	0.022 (5)	0.023 (5)	0.017 (5)	0.002 (5)	0.005 (5)	0.009 (5)
C26B	0.019 (5)	0.024 (5)	0.019 (5)	0.003 (5)	0.001 (5)	0.008 (5)
C27B	0.020 (5)	0.023 (5)	0.019 (5)	0.001 (5)	−0.001 (5)	0.006 (5)
C28B	0.016 (4)	0.019 (4)	0.016 (4)	0.000 (4)	0.000 (4)	0.004 (4)
O12B	0.015 (5)	0.018 (5)	0.018 (5)	0.000 (5)	0.008 (5)	0.003 (5)
C29	0.0243 (14)	0.0280 (15)	0.0260 (15)	−0.0008 (12)	−0.0020 (11)	0.0028 (12)
C30	0.039 (2)	0.060 (3)	0.036 (2)	0.018 (2)	−0.0092 (17)	−0.002 (2)
C31	0.037 (2)	0.0294 (18)	0.061 (3)	0.0047 (15)	0.0089 (19)	−0.0162 (19)
C32	0.0326 (15)	0.0191 (12)	0.0171 (12)	−0.0015 (11)	0.0066 (11)	0.0000 (10)
C33	0.054 (3)	0.151 (7)	0.0213 (18)	0.050 (4)	0.0117 (19)	0.013 (3)
C34	0.055 (3)	0.0336 (19)	0.0257 (17)	−0.0055 (17)	−0.0032 (16)	0.0117 (15)
C35	0.0250 (13)	0.0130 (10)	0.0245 (13)	−0.0002 (9)	0.0027 (10)	0.0010 (10)
C36	0.0292 (18)	0.066 (3)	0.041 (2)	0.0180 (19)	−0.0015 (16)	−0.012 (2)
C37	0.0275 (15)	0.0373 (18)	0.0272 (16)	−0.0010 (13)	0.0052 (12)	−0.0123 (14)
C38	0.047 (2)	0.0276 (16)	0.0217 (14)	0.0048 (15)	0.0063 (14)	0.0006 (12)
C39	0.037 (2)	0.041 (2)	0.052 (3)	0.0086 (17)	0.0178 (18)	0.024 (2)
C40	0.055 (3)	0.0300 (18)	0.042 (2)	−0.0052 (17)	0.0205 (19)	0.0083 (16)
C41	0.0292 (17)	0.0345 (19)	0.044 (2)	−0.0054 (14)	0.0054 (15)	−0.0069 (17)
C42	0.049 (3)	0.0293 (19)	0.057 (3)	−0.0010 (18)	0.005 (2)	−0.0033 (19)
C43	0.059 (3)	0.047 (3)	0.049 (3)	−0.005 (2)	0.019 (2)	0.011 (2)
N5	0.0238 (12)	0.0210 (11)	0.0321 (14)	0.0043 (9)	0.0003 (10)	−0.0048 (11)
N6	0.0300 (14)	0.0401 (16)	0.0149 (11)	−0.0027 (12)	0.0000 (10)	0.0039 (11)
N7	0.0221 (11)	0.0226 (11)	0.0246 (12)	0.0035 (9)	0.0015 (9)	−0.0048 (10)
N8	0.0342 (15)	0.0257 (13)	0.0256 (13)	−0.0023 (11)	0.0060 (11)	0.0063 (11)
N9	0.0285 (14)	0.0339 (16)	0.0392 (17)	−0.0015 (12)	0.0033 (13)	0.0031 (14)
O13	0.0217 (10)	0.0291 (12)	0.0383 (14)	0.0060 (9)	0.0000 (10)	0.0038 (11)
O14	0.0294 (11)	0.0281 (11)	0.0171 (9)	0.0044 (9)	0.0037 (8)	0.0010 (8)
O15	0.0379 (13)	0.0190 (10)	0.0226 (11)	−0.0026 (9)	0.0029 (9)	−0.0014 (8)
O16	0.0580 (18)	0.0235 (11)	0.0185 (10)	−0.0016 (11)	0.0049 (11)	0.0034 (9)
O17	0.0419 (17)	0.0467 (18)	0.068 (2)	−0.0145 (14)	0.0273 (16)	−0.0118 (17)
O18	0.0238 (10)	0.0173 (9)	0.0271 (11)	−0.0008 (8)	−0.0059 (8)	0.0037 (8)
O19	0.0211 (9)	0.0178 (9)	0.0269 (11)	0.0012 (7)	0.0051 (8)	−0.0014 (8)
Mn1	0.02177 (19)	0.01436 (16)	0.01645 (18)	0.00213 (14)	0.00120 (14)	0.00085 (14)

### Geometric parameters (Å, º)

**Table:**

Cu1—O1B	1.845 (15)	O5B—C8B	1.297 (16)
Cu1—O1B^i^	1.845 (15)	C8B—C9B	1.473 (15)
Cu1—O1^i^	1.8945 (19)	C9B—C10B	1.405 (16)
Cu1—O1	1.8945 (19)	C9B—C14B	1.422 (16)
Cu1—O4	1.897 (2)	C10B—C11B	1.381 (18)
Cu1—O4^i^	1.897 (2)	C10B—H10B	0.9500
Cu1—O4B^i^	1.899 (16)	C11B—C12B	1.412 (19)
Cu1—O4B	1.899 (16)	C11B—H11B	0.9500
Cu2—O6^i^	1.870 (2)	C12B—C13B	1.372 (18)
Cu2—O1	1.904 (2)	C12B—H12B	0.9500
Cu2—N2^i^	1.932 (3)	C13B—C14B	1.423 (17)
Cu2—O2	1.951 (2)	C13B—H13B	0.9500
O1—N1	1.408 (4)	C14B—O6B	1.334 (16)
N1—C1	1.304 (4)	Cu5B—O12B^ii^	1.86 (3)
N1—Cu3	1.918 (3)	Cu5B—O7B	1.914 (16)
O2—C1	1.300 (3)	Cu5B—O8B	1.978 (17)
C1—C2	1.484 (4)	Cu5B—N4B^ii^	1.99 (3)
C2—C3	1.407 (4)	O7B—N3B	1.418 (18)
C2—C7	1.431 (4)	N3B—C15B	1.302 (17)
C3—C4	1.391 (4)	N3B—Cu6B	1.935 (15)
C3—H3	0.9500	O8B—C15B	1.297 (17)
C4—C5	1.398 (5)	C15B—C16B	1.480 (16)
C4—H4	0.9500	C16B—C17B	1.406 (18)
C5—C6	1.385 (4)	C16B—C21B	1.421 (17)
C5—H5	0.9500	C17B—C18B	1.385 (19)
C6—C7	1.417 (4)	C17B—H17B	0.9500
C6—H6	0.9500	C18B—C19B	1.399 (19)
C7—O3	1.332 (3)	C18B—H18B	0.9500
O3—Cu3	1.892 (2)	C19B—C20B	1.373 (19)
Cu3—O4	1.920 (2)	C19B—H19B	0.9500
Cu3—O5	1.952 (2)	C20B—C21B	1.411 (18)
O4—N2	1.405 (3)	C20B—H20B	0.9500
N2—C8	1.312 (4)	C21B—O9B	1.335 (17)
O5—C8	1.311 (3)	O9B—Cu6B	1.868 (15)
C8—C9	1.469 (4)	Cu6B—O10B	1.903 (16)
C9—C10	1.407 (4)	Cu6B—O11B	1.955 (15)
C9—C14	1.425 (4)	O10B—N4B	1.413 (19)
C10—C11	1.377 (5)	N4B—C22B	1.319 (19)
C10—H10	0.9500	O11B—C22B	1.292 (18)
C11—C12	1.408 (5)	C22B—C23B	1.465 (16)
C11—H11	0.9500	C23B—C24B	1.400 (18)
C12—C13	1.377 (5)	C23B—C28B	1.419 (17)
C12—H12	0.9500	C24B—C25B	1.393 (19)
C13—C14	1.413 (4)	C24B—H24B	0.9500
C13—H13	0.9500	C25B—C26B	1.408 (19)
C14—O6	1.322 (4)	C25B—H25B	0.9500
Cu4—O10B^ii^	1.87 (2)	C26B—C27B	1.386 (19)
Cu4—O10B	1.87 (2)	C26B—H26B	0.9500
Cu4—O10^ii^	1.8908 (18)	C27B—C28B	1.424 (18)
Cu4—O10	1.8908 (18)	C27B—H27B	0.9500
Cu4—O7^ii^	1.8925 (19)	C28B—O12B	1.331 (18)
Cu4—O7	1.8926 (19)	C29—O13	1.227 (4)
Cu4—O7B^ii^	1.93 (2)	C29—N5	1.330 (4)
Cu4—O7B	1.93 (2)	C29—H29	0.9500
Cu5—O12^ii^	1.8706 (19)	C30—N5	1.432 (5)
Cu5—O7	1.9173 (19)	C30—H30A	0.9800
Cu5—N4^ii^	1.926 (2)	C30—H30B	0.9800
Cu5—O8	1.975 (2)	C30—H30C	0.9800
O7—N3	1.409 (3)	C31—N5	1.463 (5)
N3—C15	1.299 (4)	C31—H31A	0.9800
N3—Cu6	1.928 (2)	C31—H31B	0.9800
O8—C15	1.300 (3)	C31—H31C	0.9800
C15—C16	1.484 (4)	C32—O14	1.238 (4)
C16—C17	1.411 (4)	C32—N6	1.322 (4)
C16—C21	1.424 (4)	C32—H32	0.9500
C17—C18	1.384 (4)	C33—N6	1.447 (6)
C17—H17	0.9500	C33—H33A	0.9800
C18—C19	1.400 (5)	C33—H33B	0.9800
C18—H18	0.9500	C33—H33C	0.9800
C19—C20	1.377 (4)	C34—N6	1.457 (5)
C19—H19	0.9500	C34—H34A	0.9800
C20—C21	1.407 (4)	C34—H34B	0.9800
C20—H20	0.9500	C34—H34C	0.9800
C21—O9	1.338 (3)	C35—O15	1.244 (4)
O9—Cu6	1.887 (2)	C35—N7	1.313 (4)
Cu6—O10	1.9024 (19)	C35—H35	0.9500
Cu6—O11	1.9301 (19)	C36—N7	1.455 (5)
O10—N4	1.408 (3)	C36—H36A	0.9800
N4—C22	1.311 (3)	C36—H36B	0.9800
O11—C22	1.303 (3)	C36—H36C	0.9800
C22—C23	1.476 (3)	C37—N7	1.458 (4)
C23—C24	1.408 (4)	C37—H37A	0.9800
C23—C28	1.427 (4)	C37—H37B	0.9800
C24—C25	1.389 (4)	C37—H37C	0.9800
C24—H24	0.9500	C38—O16	1.247 (5)
C25—C26	1.402 (4)	C38—N8	1.321 (5)
C25—H25	0.9500	C38—H38	0.9500
C26—C27	1.385 (4)	C39—N8	1.466 (5)
C26—H26	0.9500	C39—H39A	0.9800
C27—C28	1.423 (4)	C39—H39B	0.9800
C27—H27	0.9500	C39—H39C	0.9800
C28—O12	1.321 (3)	C40—N8	1.453 (5)
Cu2B—O6B^i^	1.851 (18)	C40—H40A	0.9800
Cu2B—O1B	1.900 (12)	C40—H40B	0.9800
Cu2B—N2B^i^	1.96 (2)	C40—H40C	0.9800
Cu2B—O2B	1.972 (14)	C41—O17	1.218 (5)
O1B—N1B	1.397 (17)	C41—N9	1.351 (5)
N1B—C1B	1.305 (16)	C41—H41	0.9500
N1B—Cu3B	1.929 (13)	C42—N9	1.445 (6)
O2B—C1B	1.300 (15)	C42—H42A	0.9800
C1B—C2B	1.491 (15)	C42—H42B	0.9800
C2B—C3B	1.407 (17)	C42—H42C	0.9800
C2B—C7B	1.423 (16)	C43—N9	1.457 (6)
C3B—C4B	1.401 (19)	C43—H43A	0.9800
C3B—H3B	0.9500	C43—H43B	0.9800
C4B—C5B	1.396 (19)	C43—H43C	0.9800
C4B—H4B	0.9500	O13—Mn1	2.152 (2)
C5B—C6B	1.381 (18)	O14—Mn1	2.231 (2)
C5B—H5B	0.9500	O15—Mn1	2.146 (2)
C6B—C7B	1.410 (16)	O16—Mn1	2.150 (2)
C6B—H6B	0.9500	O18—Mn1	2.171 (2)
C7B—O3B	1.357 (15)	O18—H18C	0.826 (19)
O3B—Cu3B	1.886 (12)	O18—H18D	0.836 (19)
Cu3B—O4B	1.913 (13)	O19—Mn1	2.178 (2)
Cu3B—O5B	1.980 (12)	O19—H19C	0.834 (19)
O4B—N2B	1.413 (17)	O19—H19D	0.839 (19)
N2B—C8B	1.306 (18)		
			
O1^i^—Cu1—O1	180.0	N2B—O4B—Cu1	119 (2)
O1^i^—Cu1—O4	91.80 (8)	N2B—O4B—Cu3B	111.2 (13)
O1—Cu1—O4	88.20 (8)	Cu1—O4B—Cu3B	114.9 (8)
O1^i^—Cu1—O4^i^	88.20 (8)	C8B—N2B—O4B	113.2 (16)
O1—Cu1—O4^i^	91.80 (8)	C8B—O5B—Cu3B	108.9 (10)
O4—Cu1—O4^i^	180.0	O5B—C8B—N2B	121.9 (15)
O1^i^—Cu1—O4B^i^	50.0 (4)	O5B—C8B—C9B	117.4 (13)
O1—Cu1—O4B^i^	130.0 (4)	N2B—C8B—C9B	120.6 (15)
O4—Cu1—O4B^i^	141.7 (4)	C10B—C9B—C14B	121.4 (14)
O4^i^—Cu1—O4B^i^	38.3 (4)	C10B—C9B—C8B	115.9 (14)
O1B—Cu1—O4B	90.1 (6)	C14B—C9B—C8B	122.7 (14)
O1B^i^—Cu1—O4B	89.9 (6)	C11B—C10B—C9B	119.6 (17)
O4B^i^—Cu1—O4B	180.0 (6)	C11B—C10B—H10B	120.2
O6^i^—Cu2—O1	163.64 (11)	C9B—C10B—H10B	120.2
O6^i^—Cu2—N2^i^	92.76 (11)	C10B—C11B—C12B	120 (2)
O1—Cu2—N2^i^	90.34 (10)	C10B—C11B—H11B	119.8
O6^i^—Cu2—O2	98.90 (9)	C12B—C11B—H11B	119.8
O1—Cu2—O2	81.42 (8)	C13B—C12B—C11B	119 (2)
N2^i^—Cu2—O2	164.29 (11)	C13B—C12B—H12B	120.6
N1—O1—Cu1	117.7 (2)	C11B—C12B—H12B	120.6
N1—O1—Cu2	113.54 (16)	C12B—C13B—C14B	122.8 (19)
Cu1—O1—Cu2	117.99 (10)	C12B—C13B—H13B	118.6
C1—N1—O1	111.2 (2)	C14B—C13B—H13B	118.6
C1—N1—Cu3	130.9 (2)	O6B—C14B—C9B	126.8 (15)
O1—N1—Cu3	117.4 (2)	O6B—C14B—C13B	117.0 (15)
C1—O2—Cu2	110.62 (16)	C9B—C14B—C13B	116.1 (14)
O2—C1—N1	122.2 (3)	O12B^ii^—Cu5B—O7B	165.6 (17)
O2—C1—C2	118.7 (2)	O12B^ii^—Cu5B—O8B	98.4 (10)
N1—C1—C2	119.1 (3)	O7B—Cu5B—O8B	81.0 (8)
C3—C2—C7	119.1 (2)	N3B—O7B—Cu5B	112.8 (19)
C3—C2—C1	117.0 (3)	N3B—O7B—Cu4	116 (3)
C7—C2—C1	124.0 (2)	Cu5B—O7B—Cu4	115.0 (13)
C4—C3—C2	122.4 (3)	C15B—N3B—O7B	110.1 (14)
C4—C3—H3	118.8	C15B—N3B—Cu6B	131.7 (19)
C2—C3—H3	118.8	O7B—N3B—Cu6B	118.0 (13)
C3—C4—C5	118.5 (3)	C15B—O8B—Cu5B	108.6 (14)
C3—C4—H4	120.7	O8B—C15B—N3B	124.1 (17)
C5—C4—H4	120.7	O8B—C15B—C16B	117.9 (16)
C6—C5—C4	120.5 (3)	N3B—C15B—C16B	118.0 (17)
C6—C5—H5	119.7	C17B—C16B—C21B	121.9 (18)
C4—C5—H5	119.7	C17B—C16B—C15B	114.0 (18)
C5—C6—C7	122.1 (3)	C21B—C16B—C15B	123.6 (17)
C5—C6—H6	118.9	C18B—C17B—C16B	121 (2)
C7—C6—H6	118.9	C18B—C17B—H17B	119.6
O3—C7—C6	117.1 (2)	C16B—C17B—H17B	119.6
O3—C7—C2	125.6 (2)	C17B—C18B—C19B	117 (2)
C6—C7—C2	117.3 (2)	C17B—C18B—H18B	121.4
C7—O3—Cu3	125.70 (18)	C19B—C18B—H18B	121.4
O3—Cu3—N1	93.39 (11)	C20B—C19B—C18B	122 (2)
O3—Cu3—O4	172.99 (10)	C20B—C19B—H19B	118.9
N1—Cu3—O4	89.59 (11)	C18B—C19B—H19B	118.9
O3—Cu3—O5	96.35 (9)	C19B—C20B—C21B	122 (2)
N1—Cu3—O5	170.22 (11)	C19B—C20B—H20B	118.8
O4—Cu3—O5	80.83 (8)	C21B—C20B—H20B	118.8
N2—O4—Cu1	118.59 (16)	O9B—C21B—C20B	117.4 (19)
N2—O4—Cu3	113.29 (17)	O9B—C21B—C16B	127.0 (19)
Cu1—O4—Cu3	115.68 (10)	C20B—C21B—C16B	114.5 (18)
C8—N2—O4	111.6 (2)	C21B—O9B—Cu6B	125.3 (16)
C8—N2—Cu2^i^	130.6 (2)	O9B—Cu6B—O10B	176.4 (14)
O4—N2—Cu2^i^	117.27 (19)	O9B—Cu6B—N3B	93.3 (9)
C8—O5—Cu3	111.38 (17)	O10B—Cu6B—N3B	89.5 (9)
O5—C8—N2	120.9 (3)	O9B—Cu6B—O11B	97.0 (9)
O5—C8—C9	119.9 (2)	O10B—Cu6B—O11B	80.3 (8)
N2—C8—C9	119.1 (2)	N3B—Cu6B—O11B	169.7 (10)
C10—C9—C14	119.1 (3)	N4B—O10B—Cu4	120.5 (18)
C10—C9—C8	117.5 (3)	N4B—O10B—Cu6B	111.6 (16)
C14—C9—C8	123.4 (3)	Cu4—O10B—Cu6B	119.5 (11)
C11—C10—C9	122.1 (3)	C22B—N4B—O10B	108 (2)
C11—C10—H10	119.0	C22B—O11B—Cu6B	111.1 (14)
C9—C10—H10	119.0	O11B—C22B—N4B	118 (2)
C10—C11—C12	119.2 (3)	O11B—C22B—C23B	120.7 (18)
C10—C11—H11	120.4	N4B—C22B—C23B	119.9 (19)
C12—C11—H11	120.4	C24B—C23B—C28B	121.2 (18)
C13—C12—C11	119.6 (3)	C24B—C23B—C22B	116.4 (19)
C13—C12—H12	120.2	C28B—C23B—C22B	122.4 (18)
C11—C12—H12	120.2	C25B—C24B—C23B	122 (2)
C12—C13—C14	122.5 (3)	C25B—C24B—H24B	119.2
C12—C13—H13	118.8	C23B—C24B—H24B	119.2
C14—C13—H13	118.8	C24B—C25B—C26B	117 (2)
O6—C14—C13	116.3 (3)	C24B—C25B—H25B	121.3
O6—C14—C9	126.3 (3)	C26B—C25B—H25B	121.3
C13—C14—C9	117.4 (3)	C27B—C26B—C25B	121 (2)
C14—O6—Cu2^i^	126.59 (19)	C27B—C26B—H26B	119.4
O10B^ii^—Cu4—O10B	180.0	C25B—C26B—H26B	119.4
O10^ii^—Cu4—O10	180.0	C26B—C27B—C28B	122 (2)
O10^ii^—Cu4—O7^ii^	91.89 (8)	C26B—C27B—H27B	119.1
O10—Cu4—O7^ii^	88.11 (8)	C28B—C27B—H27B	119.1
O10^ii^—Cu4—O7	88.11 (8)	O12B—C28B—C23B	126 (2)
O10—Cu4—O7	91.89 (8)	O12B—C28B—C27B	117 (2)
O7^ii^—Cu4—O7	180.0	C23B—C28B—C27B	115.8 (19)
O10^ii^—Cu4—O7B^ii^	51.5 (5)	O13—C29—N5	124.4 (3)
O10—Cu4—O7B^ii^	128.5 (5)	O13—C29—H29	117.8
O7^ii^—Cu4—O7B^ii^	143.3 (5)	N5—C29—H29	117.8
O7—Cu4—O7B^ii^	36.7 (5)	N5—C30—H30A	109.5
O10B^ii^—Cu4—O7B	90.8 (8)	N5—C30—H30B	109.5
O10B—Cu4—O7B	89.2 (8)	H30A—C30—H30B	109.5
O7B^ii^—Cu4—O7B	180.0 (12)	N5—C30—H30C	109.5
O12^ii^—Cu5—O7	175.18 (10)	H30A—C30—H30C	109.5
O12^ii^—Cu5—N4^ii^	93.15 (9)	H30B—C30—H30C	109.5
O7—Cu5—N4^ii^	88.80 (9)	N5—C31—H31A	109.5
O12^ii^—Cu5—O8	97.86 (9)	N5—C31—H31B	109.5
O7—Cu5—O8	80.37 (8)	H31A—C31—H31B	109.5
N4^ii^—Cu5—O8	168.84 (10)	N5—C31—H31C	109.5
N3—O7—Cu4	117.88 (16)	H31A—C31—H31C	109.5
N3—O7—Cu5	113.70 (15)	H31B—C31—H31C	109.5
Cu4—O7—Cu5	117.06 (10)	O14—C32—N6	125.2 (3)
C15—N3—O7	111.4 (2)	O14—C32—H32	117.4
C15—N3—Cu6	131.4 (2)	N6—C32—H32	117.4
O7—N3—Cu6	117.10 (17)	N6—C33—H33A	109.5
C15—O8—Cu5	111.01 (17)	N6—C33—H33B	109.5
N3—C15—O8	122.0 (2)	H33A—C33—H33B	109.5
N3—C15—C16	117.7 (2)	N6—C33—H33C	109.5
O8—C15—C16	120.3 (2)	H33A—C33—H33C	109.5
C17—C16—C21	119.5 (2)	H33B—C33—H33C	109.5
C17—C16—C15	116.6 (2)	N6—C34—H34A	109.5
C21—C16—C15	123.9 (2)	N6—C34—H34B	109.5
C18—C17—C16	121.3 (3)	H34A—C34—H34B	109.5
C18—C17—H17	119.3	N6—C34—H34C	109.5
C16—C17—H17	119.3	H34A—C34—H34C	109.5
C17—C18—C19	118.8 (3)	H34B—C34—H34C	109.5
C17—C18—H18	120.6	O15—C35—N7	124.6 (3)
C19—C18—H18	120.6	O15—C35—H35	117.7
C20—C19—C18	121.0 (3)	N7—C35—H35	117.7
C20—C19—H19	119.5	N7—C36—H36A	109.5
C18—C19—H19	119.5	N7—C36—H36B	109.5
C19—C20—C21	121.4 (3)	H36A—C36—H36B	109.5
C19—C20—H20	119.3	N7—C36—H36C	109.5
C21—C20—H20	119.3	H36A—C36—H36C	109.5
O9—C21—C20	116.6 (2)	H36B—C36—H36C	109.5
O9—C21—C16	125.5 (2)	N7—C37—H37A	109.5
C20—C21—C16	117.9 (3)	N7—C37—H37B	109.5
C21—O9—Cu6	123.22 (18)	H37A—C37—H37B	109.5
O9—Cu6—O10	173.05 (10)	N7—C37—H37C	109.5
O9—Cu6—N3	91.90 (10)	H37A—C37—H37C	109.5
O10—Cu6—N3	89.69 (9)	H37B—C37—H37C	109.5
O9—Cu6—O11	97.43 (9)	O16—C38—N8	126.1 (4)
O10—Cu6—O11	81.75 (8)	O16—C38—H38	116.9
N3—Cu6—O11	168.97 (10)	N8—C38—H38	116.9
N4—O10—Cu4	118.25 (18)	N8—C39—H39A	109.5
N4—O10—Cu6	113.11 (15)	N8—C39—H39B	109.5
Cu4—O10—Cu6	117.70 (10)	H39A—C39—H39B	109.5
C22—N4—O10	110.9 (2)	N8—C39—H39C	109.5
C22—N4—Cu5^ii^	130.8 (2)	H39A—C39—H39C	109.5
O10—N4—Cu5^ii^	117.82 (16)	H39B—C39—H39C	109.5
C22—O11—Cu6	110.87 (16)	N8—C40—H40A	109.5
O11—C22—N4	121.8 (2)	N8—C40—H40B	109.5
O11—C22—C23	119.4 (2)	H40A—C40—H40B	109.5
N4—C22—C23	118.9 (2)	N8—C40—H40C	109.5
C24—C23—C28	119.7 (2)	H40A—C40—H40C	109.5
C24—C23—C22	116.7 (2)	H40B—C40—H40C	109.5
C28—C23—C22	123.6 (2)	O17—C41—N9	124.4 (4)
C25—C24—C23	122.2 (3)	O17—C41—H41	117.8
C25—C24—H24	118.9	N9—C41—H41	117.8
C23—C24—H24	118.9	N9—C42—H42A	109.5
C24—C25—C26	118.4 (3)	N9—C42—H42B	109.5
C24—C25—H25	120.8	H42A—C42—H42B	109.5
C26—C25—H25	120.8	N9—C42—H42C	109.5
C27—C26—C25	120.7 (3)	H42A—C42—H42C	109.5
C27—C26—H26	119.7	H42B—C42—H42C	109.5
C25—C26—H26	119.7	N9—C43—H43A	109.5
C26—C27—C28	122.0 (3)	N9—C43—H43B	109.5
C26—C27—H27	119.0	H43A—C43—H43B	109.5
C28—C27—H27	119.0	N9—C43—H43C	109.5
O12—C28—C27	117.0 (2)	H43A—C43—H43C	109.5
O12—C28—C23	126.1 (2)	H43B—C43—H43C	109.5
C27—C28—C23	117.0 (2)	C29—N5—C30	120.4 (3)
C28—O12—Cu5^ii^	126.64 (18)	C29—N5—C31	121.1 (3)
O6B^i^—Cu2B—O1B	172.2 (10)	C30—N5—C31	118.5 (3)
O6B^i^—Cu2B—O2B	99.0 (7)	C32—N6—C33	121.3 (3)
O1B—Cu2B—O2B	80.7 (6)	C32—N6—C34	120.6 (3)
N2B^i^—Cu2B—O2B	162.9 (14)	C33—N6—C34	117.8 (3)
N1B—O1B—Cu1	121.1 (11)	C35—N7—C36	121.0 (3)
N1B—O1B—Cu2B	115.4 (10)	C35—N7—C37	120.9 (3)
Cu1—O1B—Cu2B	122.4 (8)	C36—N7—C37	118.1 (3)
C1B—N1B—O1B	110.5 (12)	C38—N8—C40	121.7 (3)
C1B—N1B—Cu3B	133.6 (13)	C38—N8—C39	122.0 (3)
O1B—N1B—Cu3B	115.7 (11)	C40—N8—C39	116.3 (3)
C1B—O2B—Cu2B	110.5 (11)	C41—N9—C42	122.0 (4)
O2B—C1B—N1B	122.8 (14)	C41—N9—C43	120.3 (4)
O2B—C1B—C2B	119.1 (13)	C42—N9—C43	117.6 (4)
N1B—C1B—C2B	118.1 (14)	C29—O13—Mn1	134.3 (2)
C3B—C2B—C7B	119.2 (14)	C32—O14—Mn1	122.4 (2)
C3B—C2B—C1B	117.5 (14)	C35—O15—Mn1	130.7 (2)
C7B—C2B—C1B	123.2 (14)	C38—O16—Mn1	137.4 (2)
C4B—C3B—C2B	120.1 (18)	Mn1—O18—H18C	123 (3)
C4B—C3B—H3B	119.9	Mn1—O18—H18D	119 (3)
C2B—C3B—H3B	119.9	H18C—O18—H18D	117 (5)
C5B—C4B—C3B	120 (2)	Mn1—O19—H19C	115 (3)
C5B—C4B—H4B	120.0	Mn1—O19—H19D	115 (3)
C3B—C4B—H4B	120.0	H19C—O19—H19D	106 (4)
C6B—C5B—C4B	119 (2)	O15—Mn1—O16	88.50 (10)
C6B—C5B—H5B	120.6	O15—Mn1—O13	95.06 (10)
C4B—C5B—H5B	120.6	O16—Mn1—O13	92.71 (11)
C5B—C6B—C7B	122.3 (18)	O15—Mn1—O18	173.20 (10)
C5B—C6B—H6B	118.9	O16—Mn1—O18	93.28 (10)
C7B—C6B—H6B	118.9	O13—Mn1—O18	91.41 (10)
O3B—C7B—C6B	115.3 (15)	O15—Mn1—O19	87.17 (9)
O3B—C7B—C2B	126.5 (14)	O16—Mn1—O19	91.91 (11)
C6B—C7B—C2B	118.0 (14)	O13—Mn1—O19	174.92 (10)
C7B—O3B—Cu3B	126.2 (12)	O18—Mn1—O19	86.21 (9)
O3B—Cu3B—O4B	172.6 (9)	O15—Mn1—O14	85.35 (9)
O3B—Cu3B—N1B	92.2 (7)	O16—Mn1—O14	173.51 (10)
O4B—Cu3B—N1B	90.8 (7)	O13—Mn1—O14	85.78 (10)
O3B—Cu3B—O5B	95.7 (6)	O18—Mn1—O14	93.07 (9)
O4B—Cu3B—O5B	82.0 (6)	O19—Mn1—O14	89.86 (9)
N1B—Cu3B—O5B	170.3 (9)		
			
O4—Cu1—O1—N1	31.5 (3)	Cu2B—O1B—N1B—C1B	−3 (4)
O4^i^—Cu1—O1—N1	−148.5 (3)	Cu1—O1B—N1B—Cu3B	13 (4)
O4—Cu1—O1—Cu2	173.61 (13)	Cu2B—O1B—N1B—Cu3B	−178.8 (16)
O4^i^—Cu1—O1—Cu2	−6.39 (13)	Cu2B—O2B—C1B—N1B	0 (4)
Cu1—O1—N1—C1	151.2 (3)	Cu2B—O2B—C1B—C2B	178 (2)
Cu2—O1—N1—C1	7.5 (5)	O1B—N1B—C1B—O2B	1 (5)
Cu1—O1—N1—Cu3	−21.4 (5)	Cu3B—N1B—C1B—O2B	177 (3)
Cu2—O1—N1—Cu3	−165.1 (2)	O1B—N1B—C1B—C2B	−177 (3)
Cu2—O2—C1—N1	−7.2 (4)	Cu3B—N1B—C1B—C2B	−1 (5)
Cu2—O2—C1—C2	174.5 (2)	O2B—C1B—C2B—C3B	1 (4)
O1—N1—C1—O2	0.0 (6)	N1B—C1B—C2B—C3B	179 (3)
Cu3—N1—C1—O2	171.4 (3)	O2B—C1B—C2B—C7B	178 (3)
O1—N1—C1—C2	178.3 (3)	N1B—C1B—C2B—C7B	−4 (5)
Cu3—N1—C1—C2	−10.3 (7)	C7B—C2B—C3B—C4B	−7 (6)
O2—C1—C2—C3	−1.7 (4)	C1B—C2B—C3B—C4B	171 (4)
N1—C1—C2—C3	179.9 (4)	C2B—C3B—C4B—C5B	15 (8)
O2—C1—C2—C7	178.4 (3)	C3B—C4B—C5B—C6B	−12 (9)
N1—C1—C2—C7	0.1 (5)	C4B—C5B—C6B—C7B	2 (7)
C7—C2—C3—C4	−2.6 (5)	C5B—C6B—C7B—O3B	−179 (4)
C1—C2—C3—C4	177.6 (3)	C5B—C6B—C7B—C2B	6 (5)
C2—C3—C4—C5	0.0 (7)	C3B—C2B—C7B—O3B	−178 (3)
C3—C4—C5—C6	1.9 (7)	C1B—C2B—C7B—O3B	5 (5)
C4—C5—C6—C7	−1.1 (6)	C3B—C2B—C7B—C6B	−3 (4)
C5—C6—C7—O3	179.2 (3)	C1B—C2B—C7B—C6B	180 (3)
C5—C6—C7—C2	−1.5 (5)	C6B—C7B—O3B—Cu3B	−175.7 (19)
C3—C2—C7—O3	−177.5 (3)	C2B—C7B—O3B—Cu3B	−1 (4)
C1—C2—C7—O3	2.3 (4)	C7B—O3B—Cu3B—N1B	−3 (2)
C3—C2—C7—C6	3.2 (4)	C7B—O3B—Cu3B—O5B	−177 (2)
C1—C2—C7—C6	−176.9 (3)	O1B—Cu1—O4B—N2B	161 (3)
C6—C7—O3—Cu3	−175.5 (2)	O1B^i^—Cu1—O4B—N2B	−19 (3)
C2—C7—O3—Cu3	5.2 (4)	O1B—Cu1—O4B—Cu3B	26.3 (12)
C7—O3—Cu3—N1	−10.5 (3)	O1B^i^—Cu1—O4B—Cu3B	−153.7 (12)
C7—O3—Cu3—O5	168.6 (2)	Cu1—O4B—N2B—C8B	−147 (4)
O1^i^—Cu1—O4—N2	8.5 (2)	Cu3B—O4B—N2B—C8B	−10 (6)
O1—Cu1—O4—N2	−171.5 (2)	Cu1—O4B—N2B—Cu2B^i^	20 (5)
O1^i^—Cu1—O4—Cu3	148.13 (12)	Cu3B—O4B—N2B—Cu2B^i^	157 (3)
O1—Cu1—O4—Cu3	−31.87 (12)	Cu3B—O5B—C8B—N2B	14 (5)
Cu1—O4—N2—C8	152.2 (2)	Cu3B—O5B—C8B—C9B	−168.8 (19)
Cu3—O4—N2—C8	11.7 (3)	O4B—N2B—C8B—O5B	−3 (7)
Cu1—O4—N2—Cu2^i^	−20.6 (3)	Cu2B^i^—N2B—C8B—O5B	−169 (3)
Cu3—O4—N2—Cu2^i^	−161.17 (14)	O4B—N2B—C8B—C9B	180 (3)
Cu3—O5—C8—N2	−8.1 (4)	Cu2B^i^—N2B—C8B—C9B	14 (7)
Cu3—O5—C8—C9	172.4 (2)	O5B—C8B—C9B—C10B	−3 (4)
O4—N2—C8—O5	−2.1 (4)	N2B—C8B—C9B—C10B	174 (4)
Cu2^i^—N2—C8—O5	169.5 (2)	O5B—C8B—C9B—C14B	177 (3)
O4—N2—C8—C9	177.4 (3)	N2B—C8B—C9B—C14B	−5 (5)
Cu2^i^—N2—C8—C9	−11.0 (5)	C14B—C9B—C10B—C11B	−5 (5)
O5—C8—C9—C10	0.9 (4)	C8B—C9B—C10B—C11B	176 (3)
N2—C8—C9—C10	−178.6 (3)	C9B—C10B—C11B—C12B	9 (6)
O5—C8—C9—C14	−179.2 (3)	C10B—C11B—C12B—C13B	−11 (8)
N2—C8—C9—C14	1.3 (5)	C11B—C12B—C13B—C14B	9 (7)
C14—C9—C10—C11	−0.5 (5)	C10B—C9B—C14B—O6B	−175 (3)
C8—C9—C10—C11	179.5 (4)	C8B—C9B—C14B—O6B	4 (5)
C9—C10—C11—C12	−1.6 (7)	C10B—C9B—C14B—C13B	2 (4)
C10—C11—C12—C13	1.7 (9)	C8B—C9B—C14B—C13B	−179 (3)
C11—C12—C13—C14	0.3 (9)	C12B—C13B—C14B—O6B	173 (4)
C12—C13—C14—O6	177.2 (5)	C12B—C13B—C14B—C9B	−4 (5)
C12—C13—C14—C9	−2.4 (6)	C9B—C14B—O6B—Cu2B^i^	−13 (4)
C10—C9—C14—O6	−177.2 (3)	C13B—C14B—O6B—Cu2B^i^	170 (2)
C8—C9—C14—O6	2.9 (5)	Cu5B—O7B—N3B—C15B	15 (10)
C10—C9—C14—C13	2.4 (5)	Cu4—O7B—N3B—C15B	150 (6)
C8—C9—C14—C13	−177.5 (3)	Cu5B—O7B—N3B—Cu6B	−161 (5)
C13—C14—O6—Cu2^i^	−176.9 (2)	Cu4—O7B—N3B—Cu6B	−25 (9)
C9—C14—O6—Cu2^i^	2.6 (5)	Cu5B—O8B—C15B—N3B	−11 (9)
O10^ii^—Cu4—O7—N3	−171.7 (2)	Cu5B—O8B—C15B—C16B	172 (4)
O10—Cu4—O7—N3	8.3 (2)	O7B—N3B—C15B—O8B	−2 (12)
O10^ii^—Cu4—O7—Cu5	−30.35 (12)	Cu6B—N3B—C15B—O8B	173 (7)
O10—Cu4—O7—Cu5	149.65 (12)	O7B—N3B—C15B—C16B	175 (6)
Cu4—O7—N3—C15	154.1 (2)	Cu6B—N3B—C15B—C16B	−10 (14)
Cu5—O7—N3—C15	11.5 (3)	O8B—C15B—C16B—C17B	5 (7)
Cu4—O7—N3—Cu6	−23.4 (3)	N3B—C15B—C16B—C17B	−172 (7)
Cu5—O7—N3—Cu6	−165.93 (15)	O8B—C15B—C16B—C21B	177 (5)
O7—N3—C15—O8	−3.6 (4)	N3B—C15B—C16B—C21B	0 (10)
Cu6—N3—C15—O8	173.3 (2)	C21B—C16B—C17B—C18B	10 (8)
O7—N3—C15—C16	176.5 (2)	C15B—C16B—C17B—C18B	−177 (5)
Cu6—N3—C15—C16	−6.5 (5)	C16B—C17B—C18B—C19B	−1 (10)
Cu5—O8—C15—N3	−5.6 (4)	C17B—C18B—C19B—C20B	−5 (12)
Cu5—O8—C15—C16	174.2 (2)	C18B—C19B—C20B—C21B	2 (12)
N3—C15—C16—C17	170.6 (3)	C19B—C20B—C21B—O9B	175 (6)
O8—C15—C16—C17	−9.2 (4)	C19B—C20B—C21B—C16B	6 (8)
N3—C15—C16—C21	−8.2 (4)	C17B—C16B—C21B—O9B	−180 (5)
O8—C15—C16—C21	172.0 (3)	C15B—C16B—C21B—O9B	9 (8)
C21—C16—C17—C18	1.5 (5)	C17B—C16B—C21B—C20B	−13 (7)
C15—C16—C17—C18	−177.4 (3)	C15B—C16B—C21B—C20B	176 (5)
C16—C17—C18—C19	−1.0 (6)	C20B—C21B—O9B—Cu6B	−173 (4)
C17—C18—C19—C20	−1.1 (8)	C16B—C21B—O9B—Cu6B	−6 (7)
C18—C19—C20—C21	2.9 (8)	C21B—O9B—Cu6B—N3B	−2 (5)
C19—C20—C21—O9	177.3 (4)	C21B—O9B—Cu6B—O11B	178 (4)
C19—C20—C21—C16	−2.3 (6)	O7B^ii^—Cu4—O10B—N4B	11 (4)
C17—C16—C21—O9	−179.4 (3)	O7B—Cu4—O10B—N4B	−169 (4)
C15—C16—C21—O9	−0.6 (5)	O7B^ii^—Cu4—O10B—Cu6B	156 (2)
C17—C16—C21—C20	0.2 (4)	O7B—Cu4—O10B—Cu6B	−24 (2)
C15—C16—C21—C20	179.0 (3)	Cu4—O10B—N4B—C22B	−178 (4)
C20—C21—O9—Cu6	−156.7 (2)	Cu6B—O10B—N4B—C22B	34 (5)
C16—C21—O9—Cu6	22.9 (4)	Cu4—O10B—N4B—Cu5B^ii^	−34 (5)
C21—O9—Cu6—N3	−27.1 (2)	Cu6B—O10B—N4B—Cu5B^ii^	178 (2)
C21—O9—Cu6—O11	158.8 (2)	Cu6B—O11B—C22B—N4B	23 (6)
O7^ii^—Cu4—O10—N4	−28.9 (3)	Cu6B—O11B—C22B—C23B	−171 (4)
O7—Cu4—O10—N4	151.1 (3)	O10B—N4B—C22B—O11B	−37 (7)
O7^ii^—Cu4—O10—Cu6	−170.64 (12)	Cu5B^ii^—N4B—C22B—O11B	−173 (4)
O7—Cu4—O10—Cu6	9.36 (12)	O10B—N4B—C22B—C23B	156 (5)
Cu4—O10—N4—C22	−153.5 (3)	Cu5B^ii^—N4B—C22B—C23B	20 (8)
Cu6—O10—N4—C22	−10.1 (5)	O11B—C22B—C23B—C24B	4 (8)
Cu4—O10—N4—Cu5^ii^	19.0 (5)	N4B—C22B—C23B—C24B	170 (6)
Cu6—O10—N4—Cu5^ii^	162.5 (2)	O11B—C22B—C23B—C28B	−177 (5)
Cu6—O11—C22—N4	8.0 (4)	N4B—C22B—C23B—C28B	−11 (8)
Cu6—O11—C22—C23	−173.11 (19)	C28B—C23B—C24B—C25B	−10 (11)
O10—N4—C22—O11	1.2 (6)	C22B—C23B—C24B—C25B	169 (9)
Cu5^ii^—N4—C22—O11	−170.1 (3)	C23B—C24B—C25B—C26B	8 (17)
O10—N4—C22—C23	−177.7 (3)	C24B—C25B—C26B—C27B	0 (18)
Cu5^ii^—N4—C22—C23	11.0 (6)	C25B—C26B—C27B—C28B	−8 (14)
O11—C22—C23—C24	−2.4 (4)	C24B—C23B—C28B—O12B	−169 (5)
N4—C22—C23—C24	176.5 (4)	C22B—C23B—C28B—O12B	13 (9)
O11—C22—C23—C28	177.3 (2)	C24B—C23B—C28B—C27B	2 (8)
N4—C22—C23—C28	−3.8 (5)	C22B—C23B—C28B—C27B	−177 (5)
C28—C23—C24—C25	1.3 (4)	C26B—C27B—C28B—O12B	178 (6)
C22—C23—C24—C25	−179.0 (3)	C26B—C27B—C28B—C23B	7 (9)
C23—C24—C25—C26	−0.9 (5)	C23B—C28B—O12B—Cu5B^ii^	−23 (8)
C24—C25—C26—C27	0.1 (5)	C27B—C28B—O12B—Cu5B^ii^	167 (4)
C25—C26—C27—C28	0.5 (5)	O13—C29—N5—C30	−2.6 (6)
C26—C27—C28—O12	179.8 (3)	O13—C29—N5—C31	179.7 (4)
C26—C27—C28—C23	−0.1 (4)	O14—C32—N6—C33	2.0 (6)
C24—C23—C28—O12	179.3 (3)	O14—C32—N6—C34	175.9 (3)
C22—C23—C28—O12	−0.3 (4)	O15—C35—N7—C36	−0.5 (5)
C24—C23—C28—C27	−0.7 (4)	O15—C35—N7—C37	−179.5 (3)
C22—C23—C28—C27	179.6 (3)	O16—C38—N8—C40	−179.2 (4)
C27—C28—O12—Cu5^ii^	177.6 (2)	O16—C38—N8—C39	1.8 (6)
C23—C28—O12—Cu5^ii^	−2.5 (4)	O17—C41—N9—C42	3.9 (7)
O4B^i^—Cu1—O1B—N1B	156 (3)	O17—C41—N9—C43	179.9 (5)
O4B—Cu1—O1B—N1B	−24 (3)	N5—C29—O13—Mn1	153.2 (3)
O4B^i^—Cu1—O1B—Cu2B	−11.3 (14)	N6—C32—O14—Mn1	176.8 (3)
O4B—Cu1—O1B—Cu2B	168.7 (14)	N7—C35—O15—Mn1	111.5 (3)
Cu1—O1B—N1B—C1B	−171 (2)	N8—C38—O16—Mn1	−68.8 (6)

Symmetry codes: (i) −*x*+2, −*y*, −*z*+1; (ii) −*x*+1, −*y*, −*z*+1.

### Hydrogen-bond geometry (Å, º)

**Table:**

*D*—H···*A*	*D*—H	H···*A*	*D*···*A*	*D*—H···*A*
O18—H18*C*···O3	0.83 (2)	2.07 (3)	2.847 (3)	156 (5)
O18—H18*D*···O9	0.84 (2)	1.95 (2)	2.778 (3)	169 (5)
O19—H19*C*···O5	0.83 (2)	1.93 (2)	2.746 (3)	167 (5)
O19—H19*D*···O17	0.84 (2)	1.88 (2)	2.713 (4)	175 (5)
